# The long transcript of lncRNA *TMPO-AS1* promotes bone metastases of prostate cancer by regulating the CSNK2A1/DDX3X complex in Wnt/β-catenin signaling

**DOI:** 10.1038/s41420-023-01585-w

**Published:** 2023-08-05

**Authors:** Min Wang, Chi Yin, Zhengquan Wu, Xinwen Wang, Qijun Lin, Xingyu Jiang, Hong Du, Chuandong Lang, Xinsheng Peng, Yuhu Dai

**Affiliations:** 1 Department of Orthopaedic Surgery, the First Affiliated Hospital, Sun Yat-Sen University, Guangzhou, 510080 China; 2 Guangdong Provincial Key Laboratory of Orthopedics and Traumatology, Guangzhou, 510080 China; 3 Department of Experimental Research, State Key Laboratory of Oncology in Southern China, Sun Yat-sen University Cancer Center, Guangzhou, 510080 China; 4 Department of Pathology, the First People’s Hospital of Guangzhou City, Guangzhou, 510080 China; 5 Department of orthopedics, The First Affiliated Hospital of USTC, Division of Life Sciences and Medicine, University of Science and Technology of China, Hefei, 230001 China

**Keywords:** Bone metastases, Long non-coding RNAs

## Abstract

The second most common male cancer is prostate cancer (PCa), which has a high tendency for bone metastasis. Long non-coding RNAs, including *TMPO-AS1*, play a crucial role in PCa progression. However, *TMPO-AS1*’s function in PCa bone metastasis (BM) and its underlying molecular mechanisms are unclear. Herein, we found that the long transcript of *TMPO-AS1* (*TMPO-AS1*_*L*_) was upregulated in PCa tissues with bone metastasis, and overexpression of *TMPO-AS1*_*L*_ correlated with advanced clinicopathological features and reduced BM-free survival in patients with PCa. Upregulated *TMPO-AS1*_*L*_ promoted, whereas downregulated *TMPO-AS1*_*L*_ inhibited, the PCa cell bone metastatic capacity in vitro and in vivo. Mechanistically, *TMPO-AS1*_*L*_ was demonstrated to act as a scaffold, that strengthened the interaction of casein kinase 2 alpha 1 (CSNK2A1) and DEAD-box helicase 3 X-linked (DDX3X), and activated the Wnt/β-catenin signaling pathway, thus promoting BM of PCa. Moreover, upregulation of *TMPO-AS1*_*L*_ in PCa resulted from transcription elongation modulated by general transcription factor IIF subunit 2 (GTF2F2). Collectively, our study provides critical insights into the role of *TMPO-AS1*_*L*_ in PCa BM via Wnt/β-catenin signaling, identifying *TMPO-AS1*_*L*_ as a candidate marker of PCa bone metastasis prognosis and therapeutic target.

## Introduction

Worldwide, the most commonly diagnosed malignancy is prostate cancer (PCa), which is the second most frequently occurring cancer in men [1]. In recent decades, marked improvements in individualized and systemic treatment for PCa have been developed. However, PCa metastasis to bone remains a primary problem, causing significant morbidity and mortality [2]. PCa patients with bone metastasis (BM) could have complications, such as pain, pathological fracture, hypercalcemia, and nerve compression syndromes, which contribute to a poor quality of life and reduce survival [3]. Therefore, an improved grasp of the mechanisms underlying PCa BM is essential to promote the development of therapeutic targets.

Cancer development and progression significantly involve the Wnt/β-catenin signaling pathway [4]. When the pathway is activated, β-catenin stabilization and nuclear translocation are promoted, resulting in increased expression of target genes. Accumulated evidence shows that Wnt/β-catenin signaling pathway could have a pivotal function in PCa BM via various downstream effectors: (i) Activation of the epithelial to mesenchymal transition (EMT) process, which is essential for PCa cell migration and invasion from the tumor’s in situ location [5]; (ii) up-regulation of anoikis resistance to survive in circulation or the bone microenvironment [6]; and (iii) reactivation of dormant disseminated PCa cells in the bone microenvironment, which initiates proliferation and forms overt metastasis [7]. Previously, we showed that Wnt/β-catenin signaling pathway activation promoted PCa BM [8]. Consequently, inhibition of the Wnt signaling pathway could be an ideal treatment strategy against PCa BM. Although intense efforts have been made to develop Wnt-targeted therapies, the results from clinical trials showed that various Wnt signaling pathway inhibitors remained suboptimal performance [9, 10]. Hence, further understanding of Wnt/β-catenin signaling pathway activation, and the identification of novel therapeutic targets might be clinically beneficial for PCa patients with BM.

Long non-coding RNAs (lncRNAs) comprise noncoding RNA molecules with a length of 200–100,000 nucleotides. lncRNAs, lacking coding potential, encompass natural antisense transcripts, overlapping transcripts and intronic transcripts [11]. Their complex and variable secondary structures, mean that lncRNAs can ‘sponge’ microRNAs or proteins, acting as competing endogenous RNAs (ceRNAs) [12], or regulate protein–protein or protein–DNA interactions by acting as scaffolds or guides, thus regulating a variety of cancer-associated signaling networks [13, 14]. In particular, lncRNAs located in the cytoplasm have emerged as important mediators of intracellular signaling pathways. For instance, in hepatocellular carcinoma, lncRNA*-MUF* promotes EMT via the Wnt signaling pathway [15], and inhibits SMAD family member 4 (SMAD4) protein degradation in colorectal cancer cells, thereby activating the transforming growth factor beta (TGF-β) signaling pathway [16]. Notably, aberrantly expressed lncRNAs have important funcitons in tumor BM in previous studies [17, 18], and were further recognized as potential prognostic and/or therapeutic targets [19, 20]. However, lncRNAs’ biological functions in PCa BM are unknown.

Thymopoietin (TMPO)-antisense RNA 1 (*TMPO-AS1*), located on chromosome 12, has been identified as a critical lncRNA in the progression of many cancers [21, 22], including PCa [23]. In the present study, searching using the University of California Santa Cruz (UCSC) genome browser identified two transcripts of *TMPO-AS1* with different exons and lengths (long and short transcripts). We identify that the expression of the long transcript of *TMPO-AS1* (*TMPO-AS1*_*L*_) is upregulated significantly in PCa tissues with BM and is associated with poor patient prognosis. Experimentally, we reveal that *TMPO-AS1*_*L*_ acts as scaffold to strengthen the interaction between casein kinase 2 alpha 1 (CSNK2A1) and DEAD-box helicase 3 X-linked (DDX3X), and activates the Wnt/β-catenin signaling pathway, which reciprocally promotes PCa BM. Furthermore, general transcription factor IIF subunit 2 (GTF2F2) mediates transcriptional elongation of *TMPO-AS1* leading to the upregulation of *TMPO-AS1*_*L*_ expression. Clinically, the GTF2F2/*TMPO-AS1*_*L*_/β-catenin axis is verified in PCa tissues. Our study determine the clinical significance and regulatory mechanism of *TMPO-AS1*_*L*_ in PCa, providing a candidate therapeutic target for BM in PCa.

## Results

### *TMPO-AS1*_*L*_ levels are increased in bone-metastatic PCa tissues

**Fig. 1:**
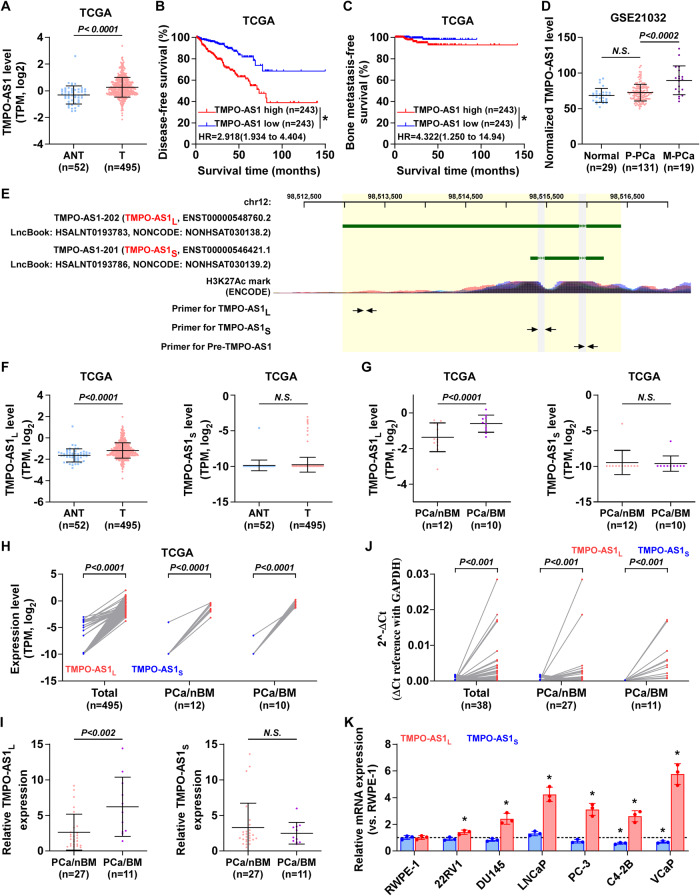
*TMPO-AS1*_*L*_ levels are increased in bone-metastatic PCa tissues. **A** *TMPO-AS1* expression in 52 adjacent normal tissues (ANT) and 495 prostate cancer tissues in the TCGA dataset. Each bar represents median values ± quartile values. *P* value was determined by *t* test. Disease‐free survival (**B**) and bone metastasis‐free survival (**C**) curves of PCa patients stratified according to *TMPO-AS1* expression in the TCGA dataset, as assessed using Kaplan–Meier analysis. *P < 0.05. **D** *TMPO-AS1* expression in ANT (*n* = 29), primary PCa tissues (p‐PCa, *n* = 131), and metastatic PCa tissues (m‐PCa, *n* = 19) in the GEO dataset (GSE21032). *P* value were determined by Kruskal-Wallis test. *N.S*. = no significance. **E** UCSC genome browser identified two *TMPO-AS1* transcripts and their exon sequences. **F** Comparison of *TMPO-AS1*_*L*_ (left panel) and *TMPO-AS1*_*S*_ (right panel) expression between ANT and PCa tissue in the TCGA dataset. *N.S*. = no significance. *P* value was determined by Mann–Whitney test. **G** Comparison of *TMPO-AS1*_*L*_ (left panel) and *TMPO-AS1*_*S*_ (right panel) expression between primary PCa tissue without bone metastasis (PCa/nBM) and primary PCa tissue with bone metastasis (PCa/BM) in the TCGA dataset. *N.S*. = no significance. *P* value was determined by Mann–Whitney test. **H** Expression comparison between *TMPO-AS1*_*L*_ and *TMPO-AS1*_*S*_ in total PCa tissue, PCa/nBM and PCa/BM in the TCGA dataset. *P* value was determined by Wilcoxon matched-pairs signed rank test. **I** qRT‐PCR analysis of *TMPO-AS1*_*L*_ (left panel) and *TMPO-AS1*_*S*_ (right panel) expression in PCa/nBM (*n* = 27) and PCa/BM (*n* = 11). Transcript levels were normalized to that of *GAPDH*. *N.S*.= no significance. *P* value was determined by Mann–Whitney test. **J** 2^-ΔCt values of *TMPO-AS1*_*L*_ and *TMPO-AS1*_*S*_ in total PCa tissue, PCa/nBM and PCa/BM by qRT‐PCR analysis relative to *GAPDH*. *P* value was determined by Wilcoxon matched-pairs signed rank test. **K** mRNA expression of *TMPO-AS1*_*L*_ and *TMPO-AS1*_*S*_ in normal prostate epithelial cell (RWPE-1), primary PCa cell 22RV1, brain metastatic cell line DU145, lymph node metastatic cell line LNCaP and three bone metastatic PCa cell lines (PC-3, C4-2B and VCaP). Transcript levels were normalized to *GAPDH*. *P < 0.05 by one-way ANOVA test.

To assess the clinical significance of *TMPO-AS1* in PCa, The Cancer Genome Atlas (TCGA) datasets were analyzed. The results showed that the expression of *TMPO-AS1* was upregulated in PCa tissues in comparison with that in adjacent normal tissues (ANT) (Fig. 1A). Survival analysis from the TCGA dataset showed that high *TMPO-AS1* expression correlated positively with lower rates of disease‐free survival (DFS) and bone metastasis-free survival (BMFS) (Fig. 1B, C). Nevertheless, for the overall survival (OS), there was no significant difference in the rates between high or low *TMPO-AS1* expression groups, which might be because the number of deaths (10 deaths in 495 cases) in the TCGA dataset was too small for meaningful analysis (Fig. S1A). The biological role of *TMPO-AS1* in the progression of PCa was assessed in the TCGA data using Gene Set Enrichment Analysis (GSEA), which revealed that high levels of *TMPO-AS1* correlated strongly and positively with metastatic propensity and EMT-associated gene signatures (Fig. S1B). This indicated that metastasis was responsible for the poor prognosis of PCa patients with high expression of *TMPO-AS1*. This was further supported by analysis of a Gene Expression Omnibus (GEO) dataset (GSE21032), which demonstrated significant upregulation of *TMPO-AS1* expression in metastatic PCa tissues (M‐PCa) in contrast to that in primary PCa tissues (P‐PCa) and ANT (Fig. 1D). PCa most commonly metastasizes to bone, therefore, we attempted to reveal the biological role of *TMPO-AS1* in PCa BM.

UCSC genome browser analysis identified two transcripts for *TMPO-AS1* that differed by their exons and lengths (*TMPO-AS1*_*L*_ and *TMPO-AS1*_*S*_) (Fig. 1E). Our results suggested that *TMPO-AS1*_*L*_ expression, rather than *TMPO-AS1*_*S*_ expression, was upregulated markedly in PCa tissues in comparison with that in ANT and in primary PCa tissue with bone metastasis (PCa/BM) compared with tissue without bone metastasis (PCa/nBM) (Fig. 1F–G). Besides, high expression of *TMPO-AS1*_*L*_, but not *TMPO-AS1*_*S*_, was associated positively with lower rates of BMFS (Fig. S1C). Meanwhile, *TMPO-AS1*_*L*_ expression was markedly higher than *TMPO-AS1*_*S*_ expression in PCa tissues (Fig. 1H). Consistently, quantitative real-time reverse transcription PCR (qRT-PCR) demonstrated that the 2^-ΔCt values for *TMPO-AS1*_*S*_ were lower than those for *TMPO-AS1*_*L*_ in PCa tissues and cell lines (Figs. 1J and S1D), and high *TMPO-AS1*_*L*_ expression was more prevalent in PCa/BM compared with that in PCa/nBM (Fig. 1I). We also found that compared with its level in the normal prostate cell line (RWPE‐1), *TMPO-AS1*_*L*_ expression was elevated in PCa cell lines (Fig. 1K). Together, these results suggest that *TMPO-AS1*_*L*_ is mainly expressed in PCa tissues and is overexpressed in bone metastatic PCa tissues.

### High *TMPO-AS1*_*L*_ expression predicts bone metastasis in prostate cancer patients

**Fig. 2:**
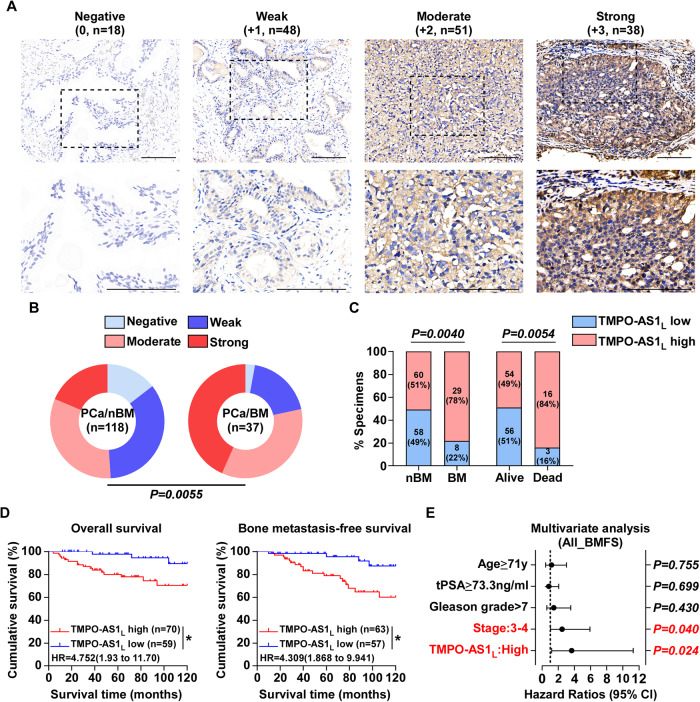
High *TMPO-AS1*_*L*_ expression predicts bone metastasis in prostate cancer patients. **A** Representative images of *TMPO-AS1*_*L*_ ISH staining in 118 PCa/nBM and 37 PCa/BM specimens. The scores were: Negative (0), weak (+ 1), moderate (+ 2) or strong (+ 3). The scores are provided in brackets. Scale bar, 100 μm. **B** ISH staining distribution for *TMPO-AS1*_*L*_ in PCa/nBM and PCa/BM patient specimens. *P* value was determined by χ2 test. **C** *TMPO-AS1*_*L*_ expression correlated significantly with patient survival and bone metastasis status. *P* value was determined by χ2 test. **D** Overall survival (OS) and bone metastasis-free survival (BMFS) in PCa patients stratified according to low and high *TMPO-AS1*_*L*_ expression as assessed using Kaplan–Meier analysis expression. *P < 0.05 by log-rank test. **E** The significance of the association between the *TMPO-AS1*_*L*_ signature and BMFS together with other important clinical variables assessed using multivariate Cox regression analysis.

In situ hybridization (ISH) was employed to detect the clinical significance of *TMPO-AS1*_*L*_ expression in 155 paraffin-embedded PCa tissues (Supplementary Table S1). The staining intensity was classified into the following degrees: Negative (0), weak (+1), moderate (+2) and strong (+3), to represent the expression levels of *TMPO-AS1*_*L*_, which was detected mainly in the cytoplasm (Fig. 2A). *TMPO-AS1*_*L*_ levels were dramatically increased in tissue from the PCa/BM compared with those in PCa/nBM (Fig. 2B), rather than *TMPO-AS1*_*S*_ (Fig. S2A, B). High *TMPO-AS1*_*L*_ levels correlated significantly with differentiation, serum prostate-specific antigen (PSA), Gleason grade, BM status, and the vital status of the patients (Fig. 2C; Supplementary Table S2). Notably, high *TMPO-AS1*_*L*_ expression was associated with shorter survival time and earlier BM (Fig. 2D). High *TMPO-AS1*_*L*_ expression was recognized as an independent prognostic factor for BMFS (Fig. 2E), but not the OS of PCa patients (Fig. S2C). These results further suggest that *TMPO-AS1*_*L*_ has a vital function in PCa BM.

### *TMPO-AS1*_*L*_ promotes PCa BM in vivo

**Fig. 3:**
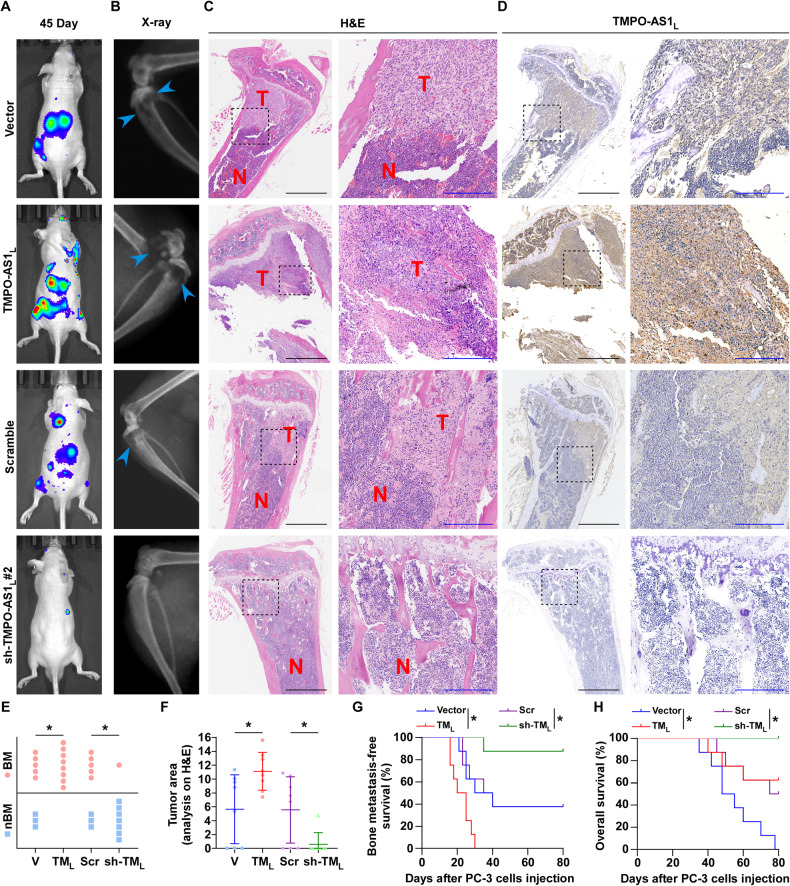
*TMPO-AS1*_*L*_ promotes bone metastasis of PC-3 cells in vivo. **A** Representative bone metastasis BLI signal in the groups of mice injected with vector, *TMPO-AS1*_*L*_‐overexpressing, scrambled, and *TMPO-AS1*_*L*_‐knockdown PC‐3 cells, respectively (*n* = 8/group; male). **B** Representative radiographical images of bone metastasis in mice from the highlighted groups (arrows point to osteolytic lesions). **C** Representative images of mouse hind limbs sections stained with H&E (T, tumor; N, the adjacent non-tumor tissues). Scar bar: black, 1 mm; blue, 250 μm. **D** Representative ISH staining for *TMPO-AS1*_*L*_ in bone and tumor lesions. Scar bar: black, 1 mm; blue, 250 μm. **E** Detected incidence of bone metastasis in the indicated groups. *P < 0.05 by χ2 test. **F** Bone osteolytic areas in the mouse hind limbs detected using histomorphometric analysis. *P < 0.05 by Mann–Whitney test. Bone metastasis‐free (**G**) and overall (**H**) survival in the indicated mouse groups according to Kaplan–Meier analysis. *P < 0.05 by long‐rank test. V, vector; TM_L_, *TMPO-AS1*_*L*_; Scr, scramble; sh-TM_L_, sh-*TMPO-AS1*_*L*_ #2.

To assess if *TMPO-AS1*_*L*_ mediates BM in PCa, we constructed a mouse model of BM, in which luciferase-labeled control PC‐3 cells, PC-3 cells with stable overexpression of *TMPO-AS1*_*L*_, and those with stably downregulated *TMPO-AS1*_*L*_ were inoculated into the nude mice’s left cardiac ventricle (Fig. S3). Bone metastatic tumor progression was monitored using BLI and X-ray imaging. Representative BLI images showed typical BM lesions in mice at day 45 in the four groups (Fig. 3A), which were also revealed through using X‐ray imaging and H&E-stained bone sections (Fig. 3B, C). Additionally, *TMPO-AS1*_*L*_ expression was identified by ISH staining in mice BM tissues (Fig. 3D). Statistical analysis of the results showed that upregulation of *TMPO-AS1*_*L*_ increased the incidence of BM, the tumor burden (H&E), and decreased mouse BMFS and OS (Fig. 3E–H). By contrast, *TMPO-AS1*_*L*_ silencing repressed the bone metastatic ability of PC-3 cells. Thus, *TMPO-AS1*_*L*_ promotes PCa bone metastasis in vivo.

### *TMPO-AS1*_*L*_ regulates multiple cell phenotypes related to bone metastasis in vitro

As documented previously, bone metastasis is a multi-step process [24]. Tumor cells need to undergo EMT progression for migration and escape from the primary site; invasion into the lymphatic system or blood vessels; survival in the circulation; homing to bone; colonization and finally flourishing in the bone environment. Every step of the process is tightly regulated by a specific set of genes [25]. To investigate whether these genes are regulated by *TMPO-AS1*_*L*_, and to understand the molecular mechanism of how *TMPO-AS1*_*L*_ promotes PCa bone metastasis, an RNA-seq experiment was carried out in PC-3 cells to determine the *TMPO-AS1*_*L*_-regulated transcriptome. As shown in Fig. S4, when *TMPO-AS1*_*L*_ was silenced, the expression of a series of genes was altered, which are involved in almost all the steps of bone metastasis, including EMT, invasion, anoikis resistance, homing to the bone, angiogenesis and proliferation (Fig. S4A). Then we carried out certain representative experiments for biological validation in vitro.

**Fig. 4:**
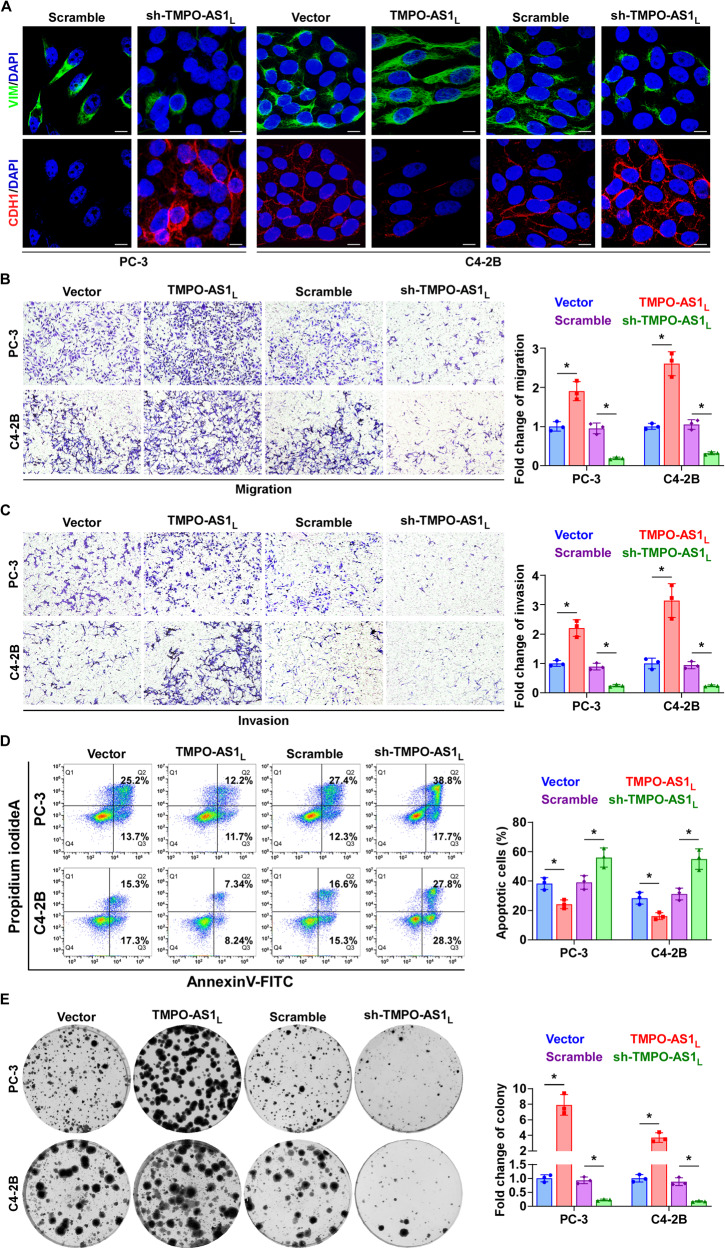
*TMPO-AS1*_*L*_ promotes EMT, migration, invasion, anoikis resistance and colony formation ability of PCa cells in vitro. **A** Immunofluorescence staining for VIM and CDH1 in PCa cells of the indicated groups. Scale bar, 10 μm. Upregulating *TMPO-AS1*_*L*_ increased, while silencing *TMPO-AS1*_*L*_ inhibited PCa cells migration (**B**) and invasion (**C**). Each bar represents the mean values ± SD of three independent experiments. *P < 0.05 by one-way ANOVA test. **D** Flow cytometry analysis of PCa cells grown in polyHEMA-coated plates for 48 h. Each bar represents the mean values ± SD of three independent experiments. *P < 0.05 by one-way ANOVA test. **E** Colony formation assay of PCa cells of the indicated groups. Each bar represents the mean values ± SD of three independent experiments. *P < 0.05 by one-way ANOVA test.

To examine the effect of *TMPO-AS1*_*L*_ on EMT in PCa cells, we analyzed the expression of the epithelial marker, CDH1 (E-cadherin) and the mesenchymal markers VIM (vimentin) and CDH2 (N-cadherin) through western blotting combined with immunofluorescence experiments. Western blotting displayed that *TMPO-AS1*_*L*_ upregulation reduced the level of CDH1 and increased levels of VIM and CDH2 in C4-2B cells; by contrast, *TMPO-AS1*_*L*_ silencing had the opposite effect on these EMT markers in both PC-3 and C4-2B cells (Fig. S4B). Notably, when *TMPO-AS1*_*L*_ expression was elevated in PC-3 cells, no significant protein level change occurred for EMT markers (Fig. S4B). We believe this is because PC-3 cells are natural mesenchymal cells, that is difficult to improve the mesenchymal cell characteristics in PC-3 cells upon *TMPO-AS1*_*L*_ overexpression. As shown in the immunofluorescence images (Fig. 4A), the protein levels of EMT markers regulated by *TMPO-AS1*_*L*_ were similar to those in the western blotting analysis, although the morphology of the PCa cells had changed. Silencing *TMPO-AS1*_*L*_ resulted in conversion of the PC-3 cell characteristic stick-like or long spindle shaped mesenchymal phenotype to an evident short spindle-shaped or cobblestone-like epithelial profile. Concurrently, PC-3 cells became confluent and formed intercellular junctions. In C4-2B cells, epithelial cell phenotypes were predominant. Therefore, *TMPO-AS1*_*L*_ converted the short spindle-shaped or cobblestone-like epithelial morphology to the stick-like or long spindle shaped mesenchymal morphology, and reduced the confluence and intercellular junctions of C4-2B cells. By contrast, silencing *TMPO-AS1*_*L*_ increased intercellular junctions (Fig. 4A).

Furthermore, we performed invasion and migration assays, anoikis resistance assays and colony formation assays. We found that *TMPO-AS1*_*L*_ overexpression enhanced the abilities of invasion, migration and colony formation in PC‐3 and C4‐2B cells, while cellular sensitivity to anoikis was decreased, whereas silencing *TMPO-AS1*_*L*_ had the opposite effects in PC‐3 and C4‐2B cells (Fig. 4B–E). In conclusion, these findings suggest that *TMPO-AS1*_*L*_ promotes PCa bone metastasis via multiple metastatic biological functions.

### *TMPO-AS1*_*L*_ activates Wnt/β-Catenin signaling

**Fig. 5:**
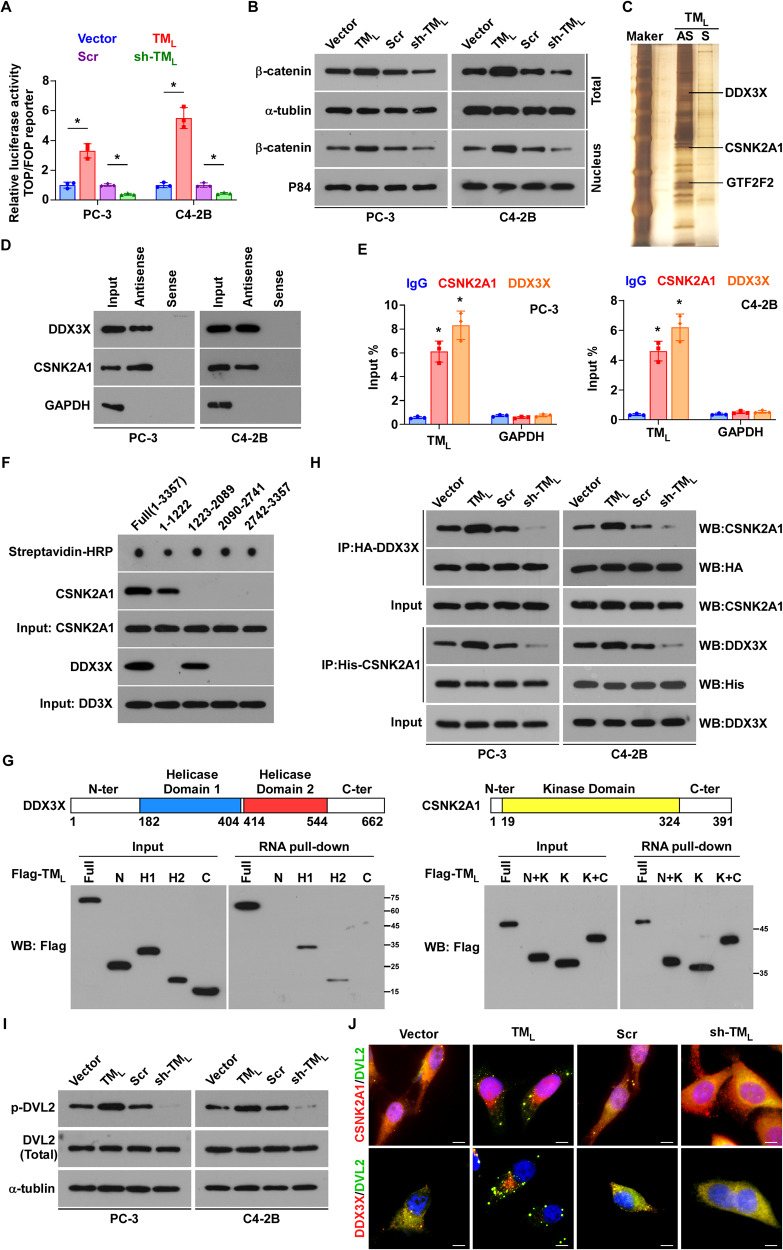
*TMPO-AS1*_*L*_ activates Wnt/β-catenin signaling and directly interacts with both CSNK2A1 and DDX3X. **A** TOP/FOP reporter activity in PCa cells of the indicated groups. Each bar represents the mean values ± SD of three independently performed experiments. *P < 0.05 by one-way ANOVA test. **B** Total and nuclear levels of β-catenin in PCa cells of the indicated groups. α-tubulin (total) and P84 (nuclear) were used loading controls. **C** Images of silver-stained gel after the RNA pull-down experiment. AS, Antisense; S, Sense. **D** RNA pull‐down and Western blotting verification of the interaction between *TMPO-AS1*_*L*_ and CSNK2A1 and DDX3X. GAPDH as a negative control. **E** The interaction of *TMPO-AS1*_*L*_ with CSNK2A1 and DDX3X, assessed using RIP with anti‐CSNK2A1, anti‐DDX3X and control IgG antibodies, followed by qRT‐PCR. The negative control was *GAPDH*. Each bar represents the mean values ± SD of three independent experiments. *P < 0.05 by one-way ANOVA test. **F** RNA pull‐down assays using full-length or truncated *TMPO-AS1*_*L*_ to identify the required core regions of *TMPO-AS1*_*L*_ for the physical interaction with CSNK2A1 and DDX3X. **G** Exogenous RNA pull-down assays showing the interaction between *TMPO-AS1*_*L*_ and different truncated mutants of DDX3X (left) and CSNK2A1 (right). The domain structures of DDX3X and CSNK2A1 are shown in the upper panel. **H** Detection of the specified proteins using co-IP in the presence of *TMPO-AS1*_*L*_ overexpression or knockdown. **I** Detection of the protein levels of total DVL2 and phospho-DVL2 (p-DVL2) in PCa cells of the indicated groups. α-tubulin were used loading controls. **J** Immunofluorescence staining for CSNK2A1, DDX3X and DVL2 in PC-3 cells of the indicated groups. Representative images showing that CSNK2A1 or DDX3X were colocalized with and DVL2 in the cytoplasm and existed as puncta. Scale bar, 10 μm. All experiments were carried out three times independently. V, vector; TM_L_, *TMPO-AS1*_*L*_; Scr, scramble; sh-TM_*L*_, sh*-TMPO-AS1*_*L*_ #2.

To investigate the molecular mechanisms of *TMPO-AS1*_*L*_ in PCa bone metastasis more deeply, we took advantage of *TMPO-AS1*_*L*_-regulated transcriptome to carry out Kyoto Encyclopedia of Genes and Genomes (KEGG) pathway analysis, which indicated that *TMPO-AS1*_*L*_ might regulate the Wnt signaling pathway (Fig. S4C). Consistent with this hypothesis, overexpressing *TMPO-AS1*_*L*_ strikingly enhanced, while silencing *TMPO-AS1*_*L*_ markedly reduced TOP/FOP luciferase activities in PCa cells (Fig. 5A). Intriguingly, almost all the *TMPO-AS1*_*L*_-regulated genes shown in Fig. S4A, encode proteins that act downstream of Wnt/β-Catenin Signaling. Further validation showed that overexpression of *TMPO-AS1*_*L*_ elevated the total and nuclear level of β-catenin, whereas knockdown of *TMPO-AS1*_*L*_ attenuated β-catenin accumulation in total cell and nuclear lysates (Fig. 5B).

In addition, we identified whether *TMPO-AS1*_*L*_ induced the biological functions of PCa cells by activating the Wnt/β-catenin pathway. XAV-939, a widely used Wnt/β-catenin inhibitor [10], abolished *TMPO-AS1*_*L*_-induced promotion of PCa cells invasion, migration, anoikis resistance and colony formation ability (Fig. S5A–D). Taken together, our findings verify that Wnt/β-catenin signaling activity is required for the *TMPO-AS1*_*L*_-induced phenotype of PCa cells.

### *TMPO-AS1*_*L*_ strengthens the interaction between CSNK2A1 and DDX3X

PhyloCSF analysis of protein‐coding potential strongly indicated that *TMPO-AS1*_*L*_ lacks protein-coding capacity (Fig. S6A). *TMPO-AS1*_*L*_ is mainly located in the cytoplasm according to RNA‐FISH assays and nuclear-cytoplasmic fractionation of PCa cells (Fig. S6B, C), which was consistent with the ISH observation in clinical PCa tissues described above (Fig. 2A). LncRNAs usually play their roles by interacting with certain proteins, therefore, we wanted to know whether specific proteins mediate Wnt/β-catenin signaling regulation by *TMPO-AS1*_*L*_. Consequently, we investigated the protein interactome of *TMPO-AS1*_*L*_ using RNA pull-down assays, and proteins that co-precipitated with the biotin-labeled antisense probe against *TMPO-AS1*_*L*_ were identified using electrophoresis through sliver-stained gels and mass spectrometry (MS) (Fig. 5C). The specific *TMPO-AS1*_*L*_-protein interactome identified using the antisense probe, was submitted to DAVID (https://david.ncifcrf.gov/home.jsp.) for KEGG pathway analysis. The proteins that interact with *TMPO-AS1*_*L*_ and regulate Wnt/β-catenin signaling from the KEGG pathway database are shown in Table S3. CSNK2A1 (unique peptides = 16), which ranked first in the table, has been well-documented as an activator of Wnt/β-catenin signaling [26]. To confirm the direct interaction between *TMPO-AS1*_*L*_ and CSNK2A1, an RNA pull-down assay followed by western blotting and RNA immunoprecipitation (RIP) assay were further performed, which verified that *TMPO-AS1*_*L*_ interacted with CSNK2A1(Fig. 5D, E).

Previous studies revealed that interactions between RNA-binding proteins (RBPs) and protein kinases could strongly enhance kinase activity [27, 28], in which process lncRNAs have been proposed to exert important function [29]. Accordingly, we speculated that certain RBPs would participate in the *TMPO-AS1*_*L*_/CSNK2A1 complex. Through screening the result of MS, combined with analyzing public databases (Starbase and BioGRID), we identified DDX3X as a suitable candidate (Fig. S6D), which was distributed in the cytoplasm and could interact with *TMPO-AS1*_*L*_ and CSNK2A1 simultaneously. RNA pull-down assay and RIP assays confirmed the direct interaction between DDX3X and *TMPO-AS1*_*L*_ (Fig. 5D, E).

Moreover, truncated forms of *TMPO-AS1*_*L*_, CSNK2A1 and DDX3X were constructed and subjected to immunoprecipitation (IP) assays to investigate binding site or domains of the three molecules. It is believed that stem-loop structures are necessary for lncRNAs to interact with proteins [30]. According to this premise, we first predicted the secondary structure of *TMPO-AS1*_*L*_ by RNAfold (http://rna.tbi.univie.ac.at/cgi-bin/RNAWebSuite/RNAfold.cgi.), and designed four fragments (1-1222 nt, 1223-2089 nt, 2090-2741 nt, and 2472-3357 nt) that retained stable stem-loop structures as shown in Fig. S7A. These fragments were transcribed and biotinylated in vitro, and then subjected to pull-down experiments using PC-3 cell extracts, which showed that fragment 1-1222 nt and fragment 1223-2089 nt of *TMPO-AS1*_*L*_, interacted with CSNK2A1 and DDX3X, respectively (Fig. 5F). Next, we sought to identify which domains of CSNK2A1 and DDX3X bind to *TMPO-AS1*_*L*_ and constructed various truncated forms of CSNK2A1 and DDX3X according to their respective domains (Fig. 5G upper panel). According to the results of RNA pull down assays, *TMPO-AS1*_*L*_ interacts with the helicase core domain of DDX3X and the protein kinase domain of CSNK2A1(Fig. 5G below panel). In addition, we found that the C-terminal domain of DDX3X was required for the interaction with CSNK2A1 via coimmunoprecipitation (co-IP) assays. Moreover, the CSNK2A1 protein kinase domain was responsible for its binding to DDX3X (Fig. S7B, C). Thus, we demonstrated the interactions among *TMPO-AS1*_*L*_, CSNK2A1 and DDX3X.

To access the role of *TMPO-AS1*_*L*_ in the RNA-protein complex, co-IP assays were employed. The results indicated that upregulated *TMPO-AS1*_*L*_ expression strengthened the interaction between CSNK2A1 and DDX3X, whereas downregulation of *TMPO-AS1*_*L*_ expression abrogated this interaction in PCa cells (Fig. 5H). Notably, we excluded the possibility of transcriptional regulation of CSNK2A1 and DDX3X by *TMPO-AS1*_*L*_ (Fig. S7D).

CSNK2A1 appears to be a multisite regulator of Wnt/β-Catenin signaling, because it can phosphorylate several components of the signaling pathway, including dishevelled segment polarity proteins (DVLs) [31] and β-catenin [32], which are the most common substrates of CSNK2A1 in the cytoplasm. As shown in Fig. 5I, the level of phosphorylated DVL2 was upregulated when *TMPO-AS1*_*L*_ was overexpressed, whereas it was downregulated when *TMPO-AS1*_*L*_ was knocked down in PCa cells. However, the level of phospho-β-catenin (Ser33/Ser37/Thr41), which would be destabilized by glycogen synthase kinase 3 beta (GSK-3β) [33], presented the opposite trend compared with phospho-DVL under the same treatments (Fig. S7E). This result indicated that *TMPO-AS1*_*L*_ enhanced the interaction between DDX3X and CSNK2A1, further increasing the activity of CSNK2A1 to phosphorylate DVL2, which activated Wnt/β-catenin signaling. Immunofluorescence experiments showed that CSNK2A1 as well as DDX3X, was colocalized with DVL2 in characteristic cytoplasmic puncta when *TMPO-AS1*_*L*_ was overexpressed. By contrast, silencing *TMPO-AS1*_*L*_ markedly reduced the number of these colocalized puncta, and CSNK2A1, DDX3X and DVL2 showed diffuse staining (Figs. 5J and S7F–G). This result indicates that *TMPO-AS1*_*L*_/CSNK2A1/DDX3X complex formation induces DVL phosphorylation by enhancing the affinity of DVL for CSNK2A1.

**Fig. 6:**
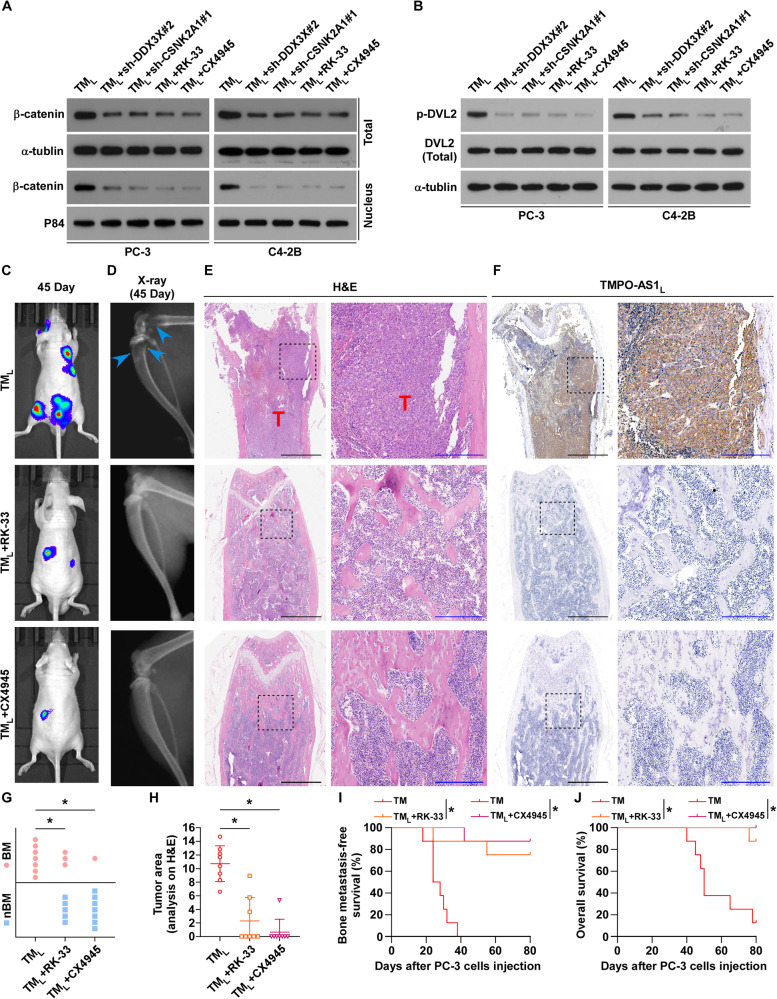
*TMPO-AS1*_*L*_ functions in a CSNK2A1/DDX3X-dependent manner. **A** β-catenin total and nuclear levels in PCa cells of the indicated groups. α-tubulin (total) and P84 (nuclear) were used as loading controls. **B** Protein levels of p-DVL2 and total DVL2 were detected in PCa cells from the indicated groups. α-tubulin were used loading controls. **C** Representative BLI signals of bone metastases in mice groups injected with PC‐3 cells overexpressing *TMPO-AS1*_*L*_ accompanied by no treatment, RK-33 or CX4945 treatment, respectively (*n* = 8/group; male). **D** Representative images of radiograph of bone metastasis of a mouse from the indicated groups (arrows indicate osteolytic lesions). **E** Representative H&E-stained sections of hind limbs from the indicated mouse (T, tumor; N, the adjacent non-tumor tissues). Scar bar: black, 1 mm; blue, 250 μm. **F** Representative ISH staining of *TMPO-AS1*_*L*_ in bone and tumor lesions. Scar bar: black, 1 mm; blue, 250 μm. **G** Bone metastasis incidence in the indicated groups. *P < 0.05 by χ2 test. **H** Hind limbs bone osteolytic areas in the indicated mice groups using histomorphometric analysis (*n* = 8/group; male). *P < 0.05 by Mann–Whitney test. BMFS (**I**) and OS (**J**) in the indicated groups (*n* = 8/group; male) according to Kaplan–Meier analysis. *P < 0.05 by the long‐rank test. RK-33 and CX4945 are inhibitors of DDX3X and CSNK2A1, respectively. TM_L_, *TMPO-AS1*_*L*_.

Finally, we carried out rescue assays to determine whether both DDX3X and CSNK2A1 are indispensable for the role of *TMPO-AS1*_*L*_ in the Wnt/β-catenin signaling and PCa BM. Silencing of DDX3X or CSNK2A1 in *TMPO-AS1*_*L*_ overexpressing PCa cells attenuated the effect of *TMPO-AS1*_*L*_ on Wnt/β-catenin activation and the phosphorylation of DVL2 (Figs. 6A, B and S8A–C). Moreover, the promotion effect of *TMPO-AS1*_*L*_-induced on cellular function in vitro and bone metastasis in vivo were also reversed by silencing DDX3X or CSNK2A1 (Figs. 6C–J and S8D–G). In addition, treatment with RK-33 and CX4945 in PCa cells, which are inhibitors of DDX3X and CSNK2A1 respectively, produced the same results in vitro and in vivo (Figs. 6A–J and S8C–G). In summary, these findings demonstrate that *TMPO-AS1*_*L*_ acts as a molecular scaffold to strengthen the interaction between DDX3X and CSNK2A1, enhancing the activity of CSNK2A1, and activating Wnt/β-catenin signaling.

### GTF2F2-mediated transcriptional elongation contributes to the upregulation of *TMPO-AS1*_*L*_ in PCa

**Fig. 7:**
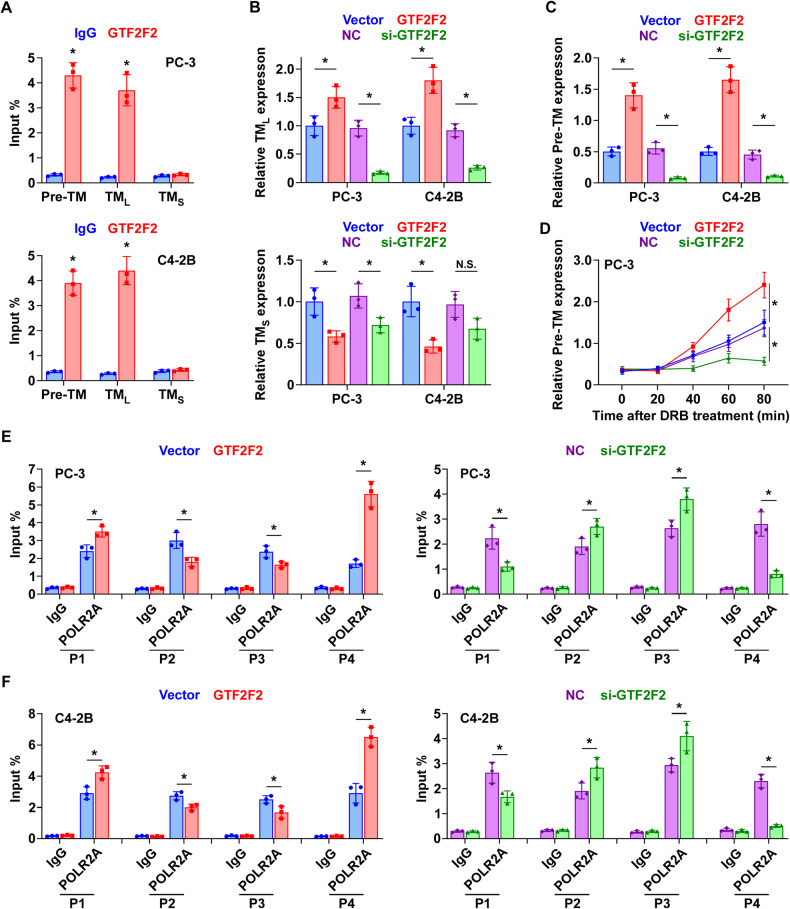
GTF2F2 participates in *TMPO-AS1*_*L*_ upregulation in PCa. **A** To examine the interaction of GTF2F2 with the pre-mRNA of *TMPO-AS1*, *TMPO-AS1*_*L*_ and *TMPO-AS1*_*S*_ in PC-3 cells (upper) and C4-2B cells (below), anti‐GTF2F2 and control IgG antibodies were used for RIP assays, with subsequent qRT‐PCR. Each bar represents the mean values ± SD of three independently performed experiments. *P < 0.05 by *t* test. qRT‐PCR analysis of *TMPO-AS1*_*L*_ (upper panel of (**B**)), *TMPO-AS1*_*S*_ (below panel of (**B**)) and the pre-mRNA of *TMPO-AS1* (**C**) expression in PCa cells of the indicated groups. Each bar represents the mean values ± SD of three independently performed experiments. *P < 0.05 by one-way ANOVA test. **D** In the specified groups, PC-3 cells were treated after 48 h with DRB at 100μM for 3 h. DRB was removed, fresh medium was supplemented, and at the indicated times, cells were collected. Images show the levels of *TMPO-AS1* pre-mRNA as assessed using qRT-PCR. Each bar represents the mean values ± SD of three independently performed experiments. *P < 0.05 by repeated measures ANOVA. The distribution of RNAPII at various positions on the gene, as detected using ChIP with anti‐POLR2A and control IgG antibodies, with subsequent qRT‐PCR analysis in PC-3 cells (**E**) and C4-2B cells (**F**) in the indicated groups. Each bar represents the mean values ± SD of three independently performed experiments. *P < 0.05 by one-way ANOVA test.

As mentioned above, the human *TMPO-AS1* gene produces two transcripts with different lengths (Fig. 1E), and the long transcript *TMPO-AS1*_*L*_ shows higher expression than the short transcript, *TMPO-AS1*_*S*_, in PCa tissues and cell lines (Figs. 1F–I and S1D). Therefore, we speculated that transcription elongation is the underlying mechanism contributing to the upregulation of *TMPO-AS1*_*L*_ in PCa cells. Interestingly, we found that GTF2F2, a subunit of transcription factor II F (TFIIF), which is a general transcription and elongation factor [34], could bind to *TMPO-AS1*_*L*_ (Fig. 5C). RIP assays validated that GTF2F2 could bind to the pre-mRNA of *TMPO-AS1* transcription products and *TMPO-AS1*_*L*_, but not *TMPO-AS1*_*S*_ (Fig. 7A). Importantly, we found that that *GTF2F2* overexpression upregulated, whereas silencing *GTF2F2* downregulated the mRNA level of *TMPO-AS1*_*L*_ and its pre-mRNA (Fig. 7B, C). Nevertheless, the level of *TMPO-AS1*_*S*_ varied irregularly, which indicated that the transcription of *TMPO-AS1*_*S*_ is incompletely modulated by GTF2F2.

The 5,6-dichloro-1-β-d-ribofuranosylbenzimidazole (RBD), a reversible transcriptional elongation inhibitor, was used to treat *GTF2F2* overexpression/knockdown PCa cells (Fig. S9A, B) for 3 h, and RNA was collected at different time points for qRT-PCR analysis to detect the level of the pre-mRNA. The pre-mRNA level before DRB treatment was used as the reference [35]. The result showed that the expression level of the pre-mRNA almost recovered at 40 min in *GTF2F2* overexpressing PCa cells, whereas it did not recover within 80 min in the *GTF2F2* knockdown PCa cells (Figs. 7D and S9C). This suggest that GTF2F2 can overcome the inhibitory effect of DRB in the transcriptional elongation process.

The distribution of RNA polymerase II (RNAPII) on a gene can reflect the dynamics of transcript elongation [36]. Therefore, we selected four sites in the *TMPO-AS1* gene, as shown in a schematic diagram (Fig. S9D): P1 (the transcription start site of *TMPO-AS1*_*L*_), P2 (the transcription start site of *TMPO-AS1*_*S*_), P3 (before the splice site) and P4 (distal region after the splice site). The specific primers corresponding to each site were designed and quantitative ChIP assays were performed under conditions of *GTF2F2* overexpression or knockdown. Overexpression of *GTF2F2* caused the recruitment of RNA Polymerase II Subunit A (POLR2A), the largest subunit of RNAPII, at P1 and P4, and decreased its distribution at P2 and P3 (Fig. 7E, F). Conversely, knockdown of *GTF2F2* induced the opposite distribution of POLR2A at the same positions (Fig. 7E, F). These findings indicate that GTF2F2 could modulate the distribution of RNAPII and the dynamics of the transcriptional elongation of the *TMPO-AS1* gene.

### Clinical relevance of the GTF2F2/*TMPO-AS1*_*L*_/β-catenin axis in clinical PCa samples

**Fig. 8:**
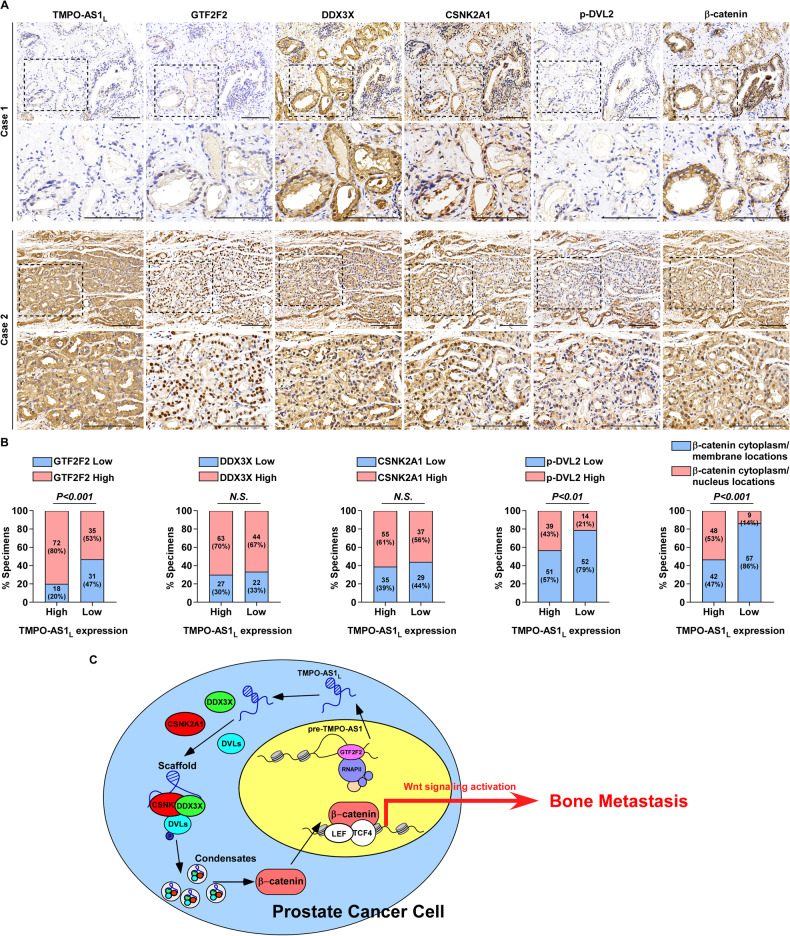
Clinical relevance of the GTF2F2/*TMPO-AS1*_*L*_/β-catenin axis in clinical PCa samples. **A** Representative images showing the expression levels of *TMPO-AS1*_*L*_, GTF2F2, DDX3X, CSNK2A1, phospho-DVL2 (p-DVL2) and β-catenin localization in clinical PCa specimens. Scale bar, 100 μm. **B** Percentage of specimens showing low or high *TMPO-AS1*_*L*_ expression in relation to the expression levels of GTF2F2, DDX3X, CSNK2A1, p-DVL2 and the cytoplasmic/nuclear or membrane levels of β-catenin. *P < 0.05 by χ2 test. **C** Schematic diagram of the regulatory mechanism of the GTF2F2/*TMPO-AS1*_*L*_/β-catenin axis in promoting PCa cell bone metastasis.

To determine the clinical impact of the GTF2F2/*TMPO-AS1*_*L*_/β-catenin axis in PCa samples, ISH and IHC assays were employed to examine the levels of GTF2F2, *TMPO-AS1*_*L*_, DDX3X, CSNK2A1, p-DVL2 and β-catenin in serial sections of PCa specimens. As shown in Fig. 8A, B, the IHC and ISH results revealed that the expression of *TMPO-AS1*_*L*_ correlated positively with GTF2F2, p-DVL2 and nuclear β-catenin levels, but not with DDX3X and CSNK2A1 levels. Overall, GTF2F2-mediated transcription elongation contributes to the upregulation of *TMPO-AS1*_*L*_ and the *TMPO-AS1*_*L*_/DDX3X/CSNK2A1 complex further activates Wnt/β-catenin signaling, thereby promoting PCa BM (Fig. 8C).

## Discussion

The present study provides critical information related to the function of *TMPO-AS1*_*L*_ in PCa BM, acting via Wnt/β-catenin signaling. Mechanistically, *TMPO-AS1*_*L*_ acts as a molecular scaffold to strengthen the interaction between DDX3X and CSNK2A1, which stimulates CSNK2A1. Increased kinase activity of CSNK2A1 elevates the levels of phosphorylated DVLs and activates Wnt/β-catenin signaling. Moreover, upregulation of *TMPO-AS1*_*L*_ in PCa results from transcription elongation modulated by GTF2F2. Importantly, high *TMPO-AS1*_*L*_ expression correlated significantly with BM in PCa patients.

Although there have been recent improvements in the individualized and systemic treatments of PCa, PCa patients with BM have reduced quality of life and survival because of BM-associated complications [2, 3]. Consequently, the identification of new therapeutic targets for PCa BM is important. The significance of their tissue-specific expression and the availability of various inhibitors make lncRNAs ideal therapeutic targets [37]. Emerging evidence supports this view: Li et al. demonstrated that inhibition of lncRNA‐MAYA repressed breast cancer BM effectively [18]; our previous study revealed that antisense oligonucleotides targeting lncRNA *PCAT6* suppressed BMs in PCa [17]. Herein, we showed that *TMPO-AS1*_*L*_ expression was associated with BM in PCa patients. Knockdown of *TMPO-AS1*_*L*_ could inhibit PCa cells BM in vivo and induced a pro-metastatic state in vitro, indicating that targeting *TMPO-AS1*_*L*_ might represent a novel and effective anti-BM treatment regimen in PCa. In agreement with these results, Huang et al. demonstrated that in PCa, *TMPO-AS1* acted an oncogenic lncRNA, and might represent a diagnostic and prognostic biomarker and a therapeutic target, for PCa [23]. However, the molecular mechanism by which *TMPO-AS1* functions was unclear. Actually, *TMPO-AS1* has been identified as an oncogene in various carcinomas [38]. Most studies demonstrated that *TMPO-AS1* might function as a miRNA sponge; however, they neglected the fact that two different transcripts are produced from the *TMPO-AS1* gene [39, 40]. In our study, we demonstrated that the long transcript *TMPO-AS1*_*L*_, rather than short transcript *TMPO-AS1*_*S*_, acted as a molecular scaffold to promote the formation of the *TMPO-AS1*_*L*_/DDX3X/CSNK2A1 RNA-protein complex, which elevated the protein kinase activity of CSNK2A1. We speculated that the sequences of *TMPO-AS1* transcripts reported in almost previous studies are similar to *TMPO-AS1*_*L*_ (*TMPO-AS1-202*, ENST00000548760.2), because the sequence of *TMPO-AS1*_*S*_ is not long enough to form a functional secondary structure. Nevertheless, we did not detect binding of AGO2 to *TMPO-AS1*_*L*_ in PCa cells using RNA pull-down assays combined with MS analysis. Thus, *TMPO-AS1*_*L*_ might not function as a ceRNA in PCa. This differs from our own results and those of other published studies about the molecular mechanisms of *TMPO-AS1*, which could be attributed to the heterogeneity of the tumor model or the context. This might explain why *TMPO-AS1* is distributed in the cytoplasm in most tumors, whereas Luo et al. found that *TMPO-AS1* was located in the nucleus in esophageal cancer, acting as transcriptional regulator [41]. Notably, our RNA pull-down and MS analysis results showed that *TMPO-AS1*_*L*_ might interact with various proteins, such as PPIP5K2, PTK2, EIF5, or others. It is possible that *TMPO-AS1*_*L*_ simultaneously exerts its biological function through multiple mechanisms in the process of BM or the progression of PCa, which should be further investigated. This reflects the diversity and complexity of the molecular mechanisms of lncRNAs.

In the current study, we found that *TMPO-AS1*_*L*_ exerted its biological role in a CSNK2A1/DDX3X-dependent manner. CSNK2A1, the catalytic subunit α of casein kinase II (CK2), is part of the tetramer of the CK2 holoenzyme or can function independently to phosphorylate substrates that have acidic residues lying C-terminal to the phosphorylated serine or threonine [26]. CSNK2A1 was certified as a pro-oncogenic factor in the majority of tumors [42], and its many substrates are components of diverse signaling pathways, such as the Wnt pathway [26], PI3K/Akt pathway [43] and NF-κB pathway [44]. CSNK2A1, which exerts multisite regulation on Wnt/β-catenin signaling, is commonly targeted to DVLs, β-catenin and TCF/LEF [44]. Considering *TMPO-AS1*_*L*_ functions in the cytoplasm, only DVLs and β-catenin are possible substrates of CSNK2A1 in our model. As shown by western blotting, *TMPO-AS1*_*L*_ upregulated the level of phosphorylated DVL, rather than phosphorylated β-catenin, which inhibits the ‘β-catenin destruction complex’, resulting in inhibition of GSK3β-mediated β-catenin phosphorylation/degradation, promoting its accumulation and translocation into the nucleus [44]. In our study, this effect of CSNK2A1 on Wnt/β-catenin signaling was modulated by DDX3X, which is defined as an RBP. Consistently, Llorens et al. reported that the RBP eIF2β binds to CSNK2A1 and affects its activity [28]. Alternatively, there is growing evidence that DDX3X interacts with protein kinases and enhances their activation, such as IKKα [45] and CSNK1E [27]. Moreover, Guan et al. revealed that the lncRNA *LINC00673-v4* regulated the protein-protein interactions between RBPs and kinases [29]. In addition, we found that *TMPO-AS1*_*L*_ promoted CSNK2A1 and DDX3X co-localization with DVLs and formed biomolecular condensates in immunofluorescence experiments. Similarly, Cruciat et al. demonstrated that the interaction between DDX3X and CSNK1E induced the recruitment of CSNK1E to DVL2 punctae [27], and further discovered that multiple DDX members also bind to and regulate the activity of the CK1 family. Based on these evidences, we speculated that interactions between RBPs and kinases occur extensively in cellular physiological and pathological processes. This interaction might be accompanied by phase separation. Schwarz-Romond et al. suggested that DVLs in the cytoplasm were involved in Wnt/β-catenin signaling, arising from a phase separation [46]; Indeed, studies have revealed that the interactions of RBPs (including DDX3X) with RNA appear to promote phase separation [47, 48]. In a model of breast cancer BM, phase separation involved CSNK2A1 in the regulation of Wnt/β-catenin signaling [49]. Taken together, these RNA/protein interactions have functional importance: phase-separated condensates might serve as reaction crucibles to facilitate efficient enzymatic reactions and sequester interference by other enzymes or signaling pathways in the cytoplasm. Despite not further investigating whether *TMPO-AS1*_*L*_/CSNK2A1/DDX3X condensates undergo phase separation, we have provided a new insight that will promote further research. Notably, Guan et al. suggested that *LINC00673-v4* enhanced lung adenocarcinoma metastasis via a similar mechanism [29]. However, *LINC00673-v4* expression was significantly lower than that of *TMPO-AS1*_*L*_ in PCa, and its expression was no significantly different between PCa/nBM and PCa/BM tissue (Fig. S1E–H). Our results suggest that this mechanism plays an important role in many tumors, but depends on diverse molecules.

Among all the studies on *TMPO-AS1*, we first distinguished the two different transcripts from the same *TMPO-AS1* gene, and found that *TMPO-AS1*_*L*_ was primarily expressed in PCa. According to the UCSC genome browser, there are at least three underlying mechanisms involving transcription of two products from the *TMPO-AS1* gene, including transcription elongation, alternative transcription start site selection and alternative splicing. We believe that transcription elongation is mainly responsible for the increased expression of *TMPO-AS1*_*L*_ in PCa, in which *TMPO-AS1*_*L*_ has a sequence long enough to form stem-loop structures. Furthermore, our results showed that GTF2F2 participated in the process of transcription elongation and upregulates the expression of *TMPO-AS1*_*L*_. GTF2F2, together with GTF2F1, constitutes the heterodimer TFIIF. TFIIF is a general transcription factor, that controls the dynamics of RNAPII at both the initiation and elongation stages of transcription [34]. This is because TFIIF is the core member of the pre-initiation-complex (PIC) and is associated with elongation complex facilitating rapid and effective transcript elongation [50]. Similarly, Marasca et al. revealed that GTF2F1 could upregulate the expression of the long transcript of RAB22A in T cells [51]. Moreover, TFIIF can promote start site selection dependent on two structured TFIIF domains and the winged helix domain of GTF2F2 [52]. Additionally, our ChIP assays showed that upregulation of GTF2F2 changed the abundance of RNAPII at the distal region after the splicing site. Whereas, knockdown GTF2F2 resulted in RNAPII stalling before the splicing site. Based on this, we speculated that the alternative exon inclusion events found in *TMPO-AS1*_*L*_ contribute to TFIIF-mediated rapid transcription elongation [53].

## Conclusion

This study investigated the biological function, molecular mechanisms and clinical implications of *TMPO-AS1*_*L*_ in PCa BM. PCa tissues with BM showed upregulated expression of *TMPO-AS1*_*L*_. Downregulation of expression inhibited bone metastasis of PCa. Mechanistically, *TMPO-AS1*_*L*_, upregulated by GTF2F2, acts as a molecular scaffold that promotes CSNK2A1’s interaction with DDX3X, subsequently activating the Wnt/β-catenin signaling pathway. These findings suggest that *TMPO-AS1*_*L*_ plays a crucial role in bone metastasis of PCa and can be a potential biomarker for the prognosis and targeted therapy for PCa patients with bone metastasis.

## Materials and methods

### Cell lines and cell culture

The normal prostate epithelial cells (RWPE-1 cells (RRID:CVCL_3791)) and the PCa cell lines (22RV1 (RRID:CVCL_1045), PC-3 (RRID:CVCL_0035), VCaP (RRID:CVCL_2235), DU145 (RRID:CVCL_0105), and LNCaP (RRID:CVCL_0395)) were provided by the Shanghai Chinese Academy of Sciences cell bank (Shanghai, China). The C4-2B cell line (RRID:CVCL_4784) was provided by the American Type Culture Collection (ATCC, Manassas, VA, USA). All cells were grown in their recommended medium supplemented with 10% fetal bovine serum (#10099141, Thermo Fisher Scientific, Waltham, MA, USA), 100 μg/ml streptomycin and 100 U/ml penicillin G at 37 °C in a humidified 5% CO_2_ atmosphere. All cells have been authenticated using short tandem repeat (STR) profiling within the last 3 years. All experiments were performed with mycoplasma-free cells.

### Mouse experiments

The Institutional Animal Care and Use Committee of Sun Yat-sen University approved the mouse experiments (Approval number: L102012019222M). To study BM, male BALB/c-nu mice aged 4–6 weeks old were anesthetized using isoflurane and then 1 × 10^6^ PC-3 cells in 100 μl of phosphate buffered saline (PBS) were inoculated into their left cardiac ventricle. Mice were included in the study if the injection into the left ventricle was successful; otherwise, they were excluded until there were 8 mice in each group. Bioluminescent imaging (BLI) was used to monitor BM, as previously described [54]. No blinding and randomization were used for the animal experiments. When the mice showed distress-like symptoms, such as 10% weight loss, paralysis, or head tilting, they were euthanized individually.

### Co-immunoprecipitation

Related plasmid DNAs were co-transfected into HEK293T cells for 48 h, rinsed two times using precooled PBS, and lysed using cold cell lysis buffer containing a protease inhibitor and phosphatase inhibitor cocktail (#11697498001, Roche, Basel, Switzerland). Next, antibodies against the target proteins were incubated with the lysates, at 4 °C overnight. Antibody-antigen complexes were captured using 50 μl of anti-HA magnetic beads or anti-His magnetic beads (#HY-K0201 or #HY-K0209, Merck, Rahway, NJ, USA) for 24 h at 4 °C. The beads were then magnetically separated and incubated with HA peptide (#HY-P0239, Merck) or His peptide (#P9811, Beyotime, Jiangsu, China) for 4 h at 4 °C. After incubation, we obtained the supernatant using magnetic separation. Both the input and Co-IP samples were analyzed by western blotting analysis using anti-HA or anti-His antibodies. Conformation-specific IgG secondary antibodies (#3678, CST, Danvers, MA, USA) were used to remove heavy- or light-chains.

### RNA pull-down assay

In vitro, a MEGAscript T7 Transcription Kit (#AM1333, Thermo Fisher Scientific) was used to transcribe *TMPO-AS1* full-length sense, antisense, and serial deletion sequences, which were subjected to biotinylation using a Pierce RNA 3′ End Desthiobiotinylation Kit (#20163, Thermo Scientific) adhering to the supplier’s guidelines, followed by RNase-free DNase I (#2270, Takara, Dalian, China) treatment and purification using an RNeasy Mini Kit (#74104, QIAGEN, Hilden, Germany). RNA pull-down assays were conducted using an RNA-Protein Pull-Down Kit (#20164, Thermo Fisher Scientific). Streptavidin-coated magnetic beads were used to capture the biotinylated RNA, which was added to cell lysates and incubated for 2 h at 4 °C. Subsequently, the beads were subjected to three washes and boiled to retrieve proteins for immunoblotting or mass spectrometry. The proteins on the sodium dodecyl sulfate-polyacrylamide gel electrophoresis gel were stained using a Pierce Silver Stain Kit (#24612, Thermo Fisher Scientific) following the supplier’s protocol.

### Statistical analyses

GraphPad Prism 9.0 (GraphPad Inc., La Jolla, CA, USA) and SPSS 19.0 (IBM Corp., Armonk, NY, USA) were used to carry out the statistical analyses. The mean ± standard deviation (SD) was used to represent the data. The quantitative experiments were carried out at least thrice independently. One way analysis of variance (ANOVA) (for normally distributed data and multiple groups) was used to analyze the quantitative data, as were the Kruskal-Wallis test (for non-normally distributed data and multiple groups), *t* tests (for normally distributed data between two groups), the Mann-Whitney U test (for non-normally distributed data between two groups), and the Wilcoxon matched-pairs signed rank test (for non-normally distributed and paired data). Categorical variables and constituent ratios were compared using the χ2 test. Kaplan–Meier curves and log-rank tests were used to analyze the survival data. Differences with a *P* < 0.05 (two-tailed) were deemed statistically significant. In figures, statistical differences are shown as follows: *N.S*. = no significance, *P < 0.05.

### Supplementary information

Supplementary Material

Western blotting datas

## Data Availability

Data are available in a public, open access repository. Data are available on reasonable request. The sequencing data and processed expression matrix have been deposited at the Sequence Read Archive database through the following accession number: SRX8803980.

## References

[1] Sung H, Ferlay J, Siegel RL, Laversanne M, Soerjomataram I, Jemal A (2021). Global Cancer Statistics 2020: GLOBOCAN estimates of incidence and mortality worldwide for 36 cancers in 185 countries. CA Cancer J Clin.

[2] Sartor O, de Bono JS (2018). Metastatic prostate cancer. N Engl J Med.

[3] Mundy GR (2002). Metastasis to bone: causes, consequences and therapeutic opportunities. Nat Rev Cancer.

[4] Clevers H (2006). Wnt/beta-catenin signaling in development and disease. Cell.

[5] Gonzalez DM, Medici D (2014). Signaling mechanisms of the epithelial-mesenchymal transition. Sci Signal.

[6] Singh AB, Sharma A, Smith JJ, Krishnan M, Chen X, Eschrich S (2011). Claudin-1 up-regulates the repressor ZEB-1 to inhibit E-cadherin expression in colon cancer cells. Gastroenterology.

[7] Giancotti FG (2013). Mechanisms governing metastatic dormancy and reactivation. Cell.

[8] Li Q, Ye L, Zhang X, Wang M, Lin C, Huang S (2017). FZD8, a target of p53, promotes bone metastasis in prostate cancer by activating canonical Wnt/beta-catenin signaling. Cancer Lett.

[9] Emami KH, Nguyen C, Ma H, Kim DH, Jeong KW, Eguchi M (2004). A small molecule inhibitor of beta-catenin/CREB-binding protein transcription [corrected]. Proc Natl Acad Sci USA.

[10] Huang SM, Mishina YM, Liu S, Cheung A, Stegmeier F, Michaud GA (2009). Tankyrase inhibition stabilizes axin and antagonizes Wnt signalling. Nature.

[11] Iyer MK, Niknafs YS, Malik R, Singhal U, Sahu A, Hosono Y (2015). The landscape of long noncoding RNAs in the human transcriptome. Nat Genet.

[12] Tay Y, Rinn J, Pandolfi PP (2014). The multilayered complexity of ceRNA crosstalk and competition. Nature.

[13] Lee S, Kopp F, Chang TC, Sataluri A, Chen B, Sivakumar S (2016). Noncoding RNA NORAD regulates genomic stability by sequestering PUMILIO proteins. Cell.

[14] Yuan JH, Yang F, Wang F, Ma JZ, Guo YJ, Tao QF (2014). A long noncoding RNA activated by TGF-beta promotes the invasion-metastasis cascade in hepatocellular carcinoma. Cancer Cell.

[15] Yan X, Zhang D, Wu W, Wu S, Qian J, Hao Y (2017). Mesenchymal stem cells promote hepatocarcinogenesis via lncRNA-MUF interaction with ANXA2 and miR-34a. Cancer Res.

[16] Wu N, Jiang M, Liu H, Chu Y, Wang D, Cao J (2021). LINC00941 promotes CRC metastasis through preventing SMAD4 protein degradation and activating the TGF-beta/SMAD2/3 signaling pathway. Cell Death Differ.

[17] Lang C, Yin C, Lin K, Li Y, Yang Q, Wu Z (2021). m(6) A modification of lncRNA PCAT6 promotes bone metastasis in prostate cancer through IGF2BP2-mediated IGF1R mRNA stabilization. Clin Transl Med.

[18] Li C, Wang S, Xing Z, Lin A, Liang K, Song J (2017). A ROR1-HER3-lncRNA signalling axis modulates the Hippo-YAP pathway to regulate bone metastasis. Nat Cell Biol.

[19] Hua JT, Ahmed M, Guo H, Zhang Y, Chen S, Soares F (2018). Risk SNP-mediated promoter-enhancer switching drives prostate cancer through lncRNA PCAT19. Cell.

[20] Mendell JT (2016). Targeting a long noncoding RNA in breast cancer. N Engl J Med.

[21] Mitobe Y, Ikeda K, Sato W, Kodama Y, Naito M, Gotoh N (2020). Proliferation-associated long noncoding RNA, TMPO-AS1, is a potential therapeutic target for triple-negative breast cancer. Cancer Sci.

[22] Zhao H, Ding F, Zheng G (2020). LncRNA TMPO-AS1 promotes LCN2 transcriptional activity and exerts oncogenic functions in ovarian cancer. FASEB J.

[23] Huang W, Su X, Yan W, Kong Z, Wang D, Huang Y (2018). Overexpression of AR-regulated lncRNA TMPO-AS1 correlates with tumor progression and poor prognosis in prostate cancer. Prostate.

[24] Leber MF, Efferth T (2009). Molecular principles of cancer invasion and metastasis (review). Int J Oncol.

[25] Krzeszinski JY, Wan Y (2015). New therapeutic targets for cancer bone metastasis. Trends Pharmacol Sci.

[26] Gao Y, Wang HY (2006). Casein kinase 2 Is activated and essential for Wnt/beta-catenin signaling. J Biol Chem.

[27] Cruciat CM, Dolde C, de Groot RE, Ohkawara B, Reinhard C, Korswagen HC (2013). RNA helicase DDX3 is a regulatory subunit of casein kinase 1 in Wnt-beta-catenin signaling. Science.

[28] Llorens F, Sarno S, Sarro E, Duarri A, Roher N, Meggio F (2005). Cross talk between protein kinase CK2 and eukaryotic translation initiation factor eIF2beta subunit. Mol Cell Biochem.

[29] Guan H, Zhu T, Wu S, Liu S, Liu B, Wu J (2019). Long noncoding RNA LINC00673-v4 promotes aggressiveness of lung adenocarcinoma via activating WNT/beta-catenin signaling. Proc Natl Acad Sci USA.

[30] Hoell JI, Larsson E, Runge S, Nusbaum JD, Duggimpudi S, Farazi TA (2011). RNA targets of wild-type and mutant FET family proteins. Nat Struct Mol Biol.

[31] Willert K, Brink M, Wodarz A, Varmus H, Nusse R (1997). Casein kinase 2 associates with and phosphorylates dishevelled. EMBO J.

[32] Song DH, Sussman DJ, Seldin DC (2000). Endogenous protein kinase CK2 participates in Wnt signaling in mammary epithelial cells. J Biol Chem.

[33] Yost C, Torres M, Miller JR, Huang E, Kimelman D, Moon RT (1996). The axis-inducing activity, stability, and subcellular distribution of beta-catenin is regulated in Xenopus embryos by glycogen synthase kinase 3. Genes Dev.

[34] Tan S, Conaway RC, Conaway JW (1995). Dissection of transcription factor TFIIF functional domains required for initiation and elongation. Proc Natl Acad Sci USA.

[35] Singh J, Padgett RA (2009). Rates of in situ transcription and splicing in large human genes. Nat Struct Mol Biol.

[36] Montes M, Cloutier A, Sanchez-Hernandez N, Michelle L, Lemieux B, Blanchette M (2012). TCERG1 regulates alternative splicing of the Bcl-x gene by modulating the rate of RNA polymerase II transcription. Mol Cell Biol.

[37] Haussecker D (2008). The business of RNAi therapeutics. Hum Gene Ther.

[38] Zheng Q, Jia J, Zhou Z, Chu Q, Lian W, Chen Z (2021). The emerging role of thymopoietin-antisense RNA 1 as long noncoding RNA in the pathogenesis of human cancers. DNA Cell Biol.

[39] Chang H, Yao Y (2022). lncRNA TMPO antisense RNA 1 promotes the malignancy of cholangiocarcinoma cells by regulating let-7g-5p/ high-mobility group A1 axis. Bioengineered.

[40] Ye J, Yan Y, Xin L, Liu J, Tang T, Bao X (2022). Long non-coding RNA TMPO-AS1 facilitates the progression of colorectal cancer cells via sponging miR-98-5p to upregulate BCAT1 expression. J Gastroenterol Hepatol.

[41] Luo XJ, He MM, Liu J, Zheng JB, Wu QN, Chen YX (2022). LncRNA TMPO-AS1 promotes esophageal squamous cell carcinoma progression by forming biomolecular condensates with FUS and p300 to regulate TMPO transcription. Exp Mol Med.

[42] Borgo C, D'Amore C, Sarno S, Salvi M, Ruzzene M (2021). Protein kinase CK2: a potential therapeutic target for diverse human diseases. Signal Transduct Target Ther.

[43] Ruzzene M, Bertacchini J, Toker A, Marmiroli S (2017). Cross-talk between the CK2 and AKT signaling pathways in cancer. Adv Biol Regul.

[44] Dominguez I, Sonenshein GE, Seldin DC (2009). Protein kinase CK2 in health and disease: CK2 and its role in Wnt and NF-kappaB signaling: linking development and cancer. Cell Mol Life Sci.

[45] Fullam A, Gu L, Hohn Y, Schroder M (2018). DDX3 directly facilitates IKKalpha activation and regulates downstream signalling pathways. Biochem J.

[46] Schwarz-Romond T, Merrifield C, Nichols BJ, Bienz M (2005). The Wnt signalling effector Dishevelled forms dynamic protein assemblies rather than stable associations with cytoplasmic vesicles. J Cell Sci.

[47] Li P, Banjade S, Cheng HC, Kim S, Chen B, Guo L (2012). Phase transitions in the assembly of multivalent signalling proteins. Nature.

[48] Shen H, Yanas A, Owens MC, Zhang C, Fritsch C, Fare CM (2022). Sexually dimorphic RNA helicases DDX3X and DDX3Y differentially regulate RNA metabolism through phase separation. Mol Cell.

[49] Esposito M, Fang C, Cook KC, Park N, Wei Y, Spadazzi C (2021). TGF-beta-induced DACT1 biomolecular condensates repress Wnt signalling to promote bone metastasis. Nat Cell Biol.

[50] Luse DS, Spangler LC, Ujvari A (2011). Efficient and rapid nucleosome traversal by RNA polymerase II depends on a combination of transcript elongation factors. J Biol Chem.

[51] Marasca F, Sinha S, Vadala R, Polimeni B, Ranzani V, Paraboschi EM (2022). LINE1 are spliced in non-canonical transcript variants to regulate T cell quiescence and exhaustion. Nat Genet.

[52] Eichner J, Chen HT, Warfield L, Hahn S (2010). Position of the general transcription factor TFIIF within the RNA polymerase II transcription preinitiation complex. EMBO J.

[53] Giono LE, Kornblihtt AR (2020). Linking transcription, RNA polymerase II elongation and alternative splicing. Biochem J.

[54] Dai Y, Ren D, Yang Q, Cui Y, Guo W, Lai Y (2017). The TGF-beta signalling negative regulator PICK1 represses prostate cancer metastasis to bone. Br J Cancer.

