# Supplementary material for: The long transcript of lncRNA TMPO-AS1 promotes bone metastases of prostate cancer by regulating the CSNK2A1/DDX3X complex in Wnt/β-catenin signaling
Source: Cell Death Discov. 2023 Aug 5;9:287. doi: 10.1038/s41420-023-01585-w (PMC10403548; doi:10.1038/s41420-023-01585-w)
Supplement: Supplementary file 1 — Supplementary Material [file 41420_2023_1585_MOESM1_ESM.docx]

**Methods**

**RNA extraction, reverse transcription, and real-time PCR**

Total RNA was extracted from tissues and cells using TRIzol reagent (Invitrogen, USA) according to the manufacturer's instructions. mRNAs and lncRNAs were reverse transcribed from total mRNA using the riboSCRIPTTM Reverse Transcription Kit (RiboBio, China). The cDNA was amplified and quantified on the CFX96 system (Bio-Rad, USA) using iQ SYBR Green Supermix (Bio-Rad, USA). The detailed information of primers is presented in Table S1, and primers for lncRNAs were synthesized and purified by RiboBio (Guangzhou, China). Real-time PCR was then conducted using SYBR Green master mix on the ViiA7 real-time PCR system (Applied Biosystems). Glyceraldehyde-3-phosphate dehydrogenase (GAPDH) was used as the endogenous control. Relative fold expressions were calculated using the comparative threshold cycle method. All real-time PCR primers are listed in online supplemental Table S4.

**Transient transfection and generation of stably transfected cell lines**

Human *TMPO-AS1* was synthesized by Obio (Shanghai, China) and cloned into the lentiviral plasmid pSin-EF2-puromycin (Addgene, Cambridge, USA). The short hairpin RNA (shRNA) against *TMPO-AS1*，DDX3X and CSNK2A1 were cloned into the pLKO.1 vector (Addgene, USA). The sequences of the separated shRNA fragments were as follows:*sh-TMPO-AS1*#1,5’-ACCTCTTCATCTCCAACAA-3’.*sh-TMPO-AS1*#2,5’-GCAATGGTATTAAGCTCAA-3’.sh-*DDX3X*#1,5’-CGGAGTGATTACGATGGCATT-3’.sh-*DDX3X*#2,5’-CGTAGAATAGTCGAACAAGAT-3’.sh-*CSNK2A1*#1,5’-ATTACCTGCAGGTGGAATATT-3’.sh-*CSNK2A1*#2,5’-TGGAATATTTCATGGACAAAT-3’.si-*GTF2F2*#1,5’-CAAAGCAACCTGTGGTGTACCTGAA-3’.si-*GTF2F2*#2,5’-CAGAACACAGGAGTGTGGCTAGTCA-3’ (synthesized by Invitrogen). The Top/Fop Flash luciferase reporter plasmid and the Renilla pRL-TK plasmids (Clontech, Japan) were used to quantitatively assess the transcriptional activity of Wnt signaling components. Transfection of plasmids was performed using Lipofectamine 3000 reagent (Invitrogen, USA). Stable cell lines expressing *TMPO-AS1*, sh-*DDX3X*, sh-*CSNK2A1* or *sh-TMPO-AS1* were generated by filtered-lentivirus infection using HEK293T cells, and selected with 0.5 mg/L puromycin (Sigma Aldrich, USA) for 10 days, as described previously.

**Western blotting**

Total protein from PCa tissues and cells was extracted with RIPA lysis buffer containing a protease inhibitor. The protein concentration was detected using a BCA kit (#P0012S, Beyotime) according to the instructions. Western blotting was performed as described previously(1). Antibodies against CDH-1 (#14472), CDH-2 (#13116), Vimentin (#5741), α-Tubulin (#3873), β-Catenin (#8480), His-Tag (#12698), HA-Tag (#3724), Flag-Tag (#147793)，Streptavidin-HRP (#3999) were purchased from Cell Signaling Technology. Antibodies against DDX3X (#ab235940), CSNK2A1 (#ab236664), GTF2F2 (#ab128945), P84 (#ab131268) were purchased from Abcam, anti-DVL2 (#MA5-26823) was purchased from Thermo Scientific and anti-p-DVL2 (#bs-13004R) was purchased from Bioss.

**Immunohistochemistry and** **in situ hybridization assays**

The immunohistochemistry and in situ hybridization assays were performed as described previously(2). Antibody information is detailed in the method section of Western blotting. The biotin-labeled *TMPO-AS1* probe (sequence:5′-AGCACCTCACGGCTCGCAACCGGCCGCCGCTCCCCGCCGGCACCACAGCAA-3′, Exons biological technology, Guangzhou, China) was used to assess *TMPO-AS1* expression by ISH staining in paraffin-embedded PCa tissues or xenograft tumors. The slides were stained with the DAB Enhanced Liquid Substrate System (#D3939, Merck). The staining score was defined as the product of the proportion of positive tumor cells score and staining intensity score. The proportion of positive tumor cells was assessed using the following criteria: 0 (no positive tumor cells); 1 (<10% positive tumor cells); 2 (10–35% positive tumor cells); 3 (35–70% positive tumor cells) and 4 (>70% positive tumor cells). The staining intensity was assessed by the following criteria: 0 (negative); 1 (weakly positive); 2 (moderately positive); 3 (strongly positive). Based on the staining score, we defined target gene expression as follows: negative staining (0 score), weak staining (1, 2, 3 and 4 scores), moderate staining (6 and 8 scores), strong staining (9 and 12 scores). The staining score was assigned by two independent pathologists for comparative evaluation of the expression of the target lncRNA or proteins. Using the highest combined sensitivity and specificity concerning patient survival, an SI score of ≥ 6 was adopted as the optimal cut-off value to define tumors with high gene expression.

**Immunofluorescence staining and RNA-fluorescence in situ hybridization**

For immunofluorescence (IF), adherent prostate cells were rinsed gently with PBS twice and fixed in 4% paraformaldehyde for 10 min at room temperature, permeabilized with 0.1% Triton for 20 min and blocked in 5% goat serum for 30 min.They were then incubated with the indicated primary antibody overnight at 4 °C. The following day, cells were incubated with the appropriate Alexa fluorochrome-conjugated secondary antibodies. The nuclei were stained with DAPI. RNA-fluorescence in situ hybridization (FISH) assays were performed using a lncRNA FISH Kit (#C10910, RiboBio) according to the manufacturer's instructions. Briefly, prostate cells were fixed, permeabilized and blocked as described for IF. Next, *TMPO-AS1* probes were added, and hybridization was performed in a dark humidified chamber at 37°C for 16 h. All images were acquired using a Nikon Eclipse Ti fluorescence microscope equipped with a Nikon DS-Qi2 monochrome camera and the NIS-Elements Viewer (5.21.00) software (Nikon).

**Transwell migration and invasion assays**

In invasion assays, diluted Matrigel matrix (#356231, Corning) was coated onto transwell chambers (#3422, Corning) firstly. The subsequent procedure was the same as that in migration assays. To evaluate migration, PCa cells were harvested after serum-free starvation for 12 h, and then resuspended in RPMI-1640 without serum. 2×10^4^ cells were seeded into the top chamber of the transwells, and the bottom chambers were filled with 500μl RPMI-1640 supplemented with 10% fetal calf serum (FBS). After incubation for 24 h, cells migrating or invading to the bottom side of the chamber were fixed with methanol and stained with 0.2% crystal violet. Three fields on one membrane (three membranes were included in every group) were selected to count migrated/invaded cells under the microscope (x100) and the average was taken as the final number of migrated/invaded cells in the indicated group.

**Colony formation assay**

The colony formation assay was performed to examine cell viability. The indicated cells (1×10^3^) were seeded in 6-well plates. Two weeks later, cells were fixed with 4% paraformaldehyde and stained with 0.2% crystal violet. Clonogenic ability was determined by counting the number of colonies.

**Anoikis resistance assay**

Anoikis resistance assay was measured by flow cytometry using the Annexin V-FITC/PI Apoptosis Detection Kit (#A211, Vazyme) according to the manufacturer's instructions. PCa cells were cultured in ultra-low attachment plates for 72 h, then harvested and centrifuged at 1000 × g for 5 min at 4°C, subsequently washed twice with pre-chilled PBS. Cells were resuspended with 100μl 1 × binding buffer and then incubated with 5µL Annexin V-FITC and 5µL Propidium iodide (PI) Staining Solution for 10 min at room temperature. Afterwards, samples were analyzed by flow cytometry within one hour after adding 400μl 1 × binding buffer.

**Patients and tumor tissues**

A total of 205 archived PCa tissues, including 165 non-bone metastatic PCa tissues and 40 bone metastatic PCa tissues were obtained during surgery or needle biopsy at the First People’s Hospital of Guangzhou City (Guangzhou, China) between January 2008 and October 2016. Patients were diagnosed on the basis of clinical and pathological evidence, and the specimens were immediately snap-frozen and stored in liquid nitrogen tanks. For the use of patients’ clinical materials for research purposes, prior patient’ consents and approval from the Institutional Research Ethics Committee were obtained. The patients’ clinicopathological features are summarized in Table S3. The medians of *TMPO-AS1*, GTF2F2, DDX3X, CSNK2A1, p-DVL2 and β-catenin expression in PCa tissues were applied to determine the corresponding protein expression levels.

**RNA immunoprecipitation and** **Chromatin immunoprecipitation**

The RNA immunoprecipitation (RIP) assay was conducted by using the EZ-Magna RIP kit (#17-109, Merck ) according to the manufacturer’s protocol. Briefly, 2×10^7^ prostate cells were lysed by RIPA lysis buffer (#20-188，Merck) containing a protease inhibitor cocktail and an RNase inhibitor. Ten percent of the lysate was saved as preimmunoprecipitation input. After washing three times with RIP buffer, beads were resuspended in 500μl RIP buffer and incubated with 5μg of the primary antibody of GTF2F2 (#ab28180, Abcam) or nonimmune species-matched IgG (#3900, CST) at 4°C for 8 h, then centrifuged at 3000 RPM for 2 min to remove supernatants. Three hundred microliters of cell lysate were added into the treated beads mentioned above overnight at 4 ℃. IgG was applied as a negative control. The immunoprecipitated RNAs were then eluted and detected by qRT-PCR as described previously. The Chromatin immunoprecipitation (ChIP) assay was performed using the SimpleChIP Enzymatic Chromatin IP Kit (#9003, CST). All procedures were performed according to the manufacturer's instructions. Briefly, prostate cancer cells (4×10^6^) were cultured in a 100 mm culture dish and transfected with the GTF2F2 plasmid or siGTF2F2. Formaldehyde was added to the culture medium to a final concentration of 1% for 10 min at room temperature for cross-linking between proteins and DNA, followed by addition of 125 mM glycine and incubation for 5 min at room temperature. Then, the indicated cells were lysed in SDS buffer, sonicated and incubated with 5μg of anti-POLRA2 antibody (#NB200-598, Novus) or anti-IgG antibody overnight at 4°C with constant rotation. The DNA fragment sizes of 250­–1,000 bp were evaluated by electrophoresis through a 1% agarose gel. The next day, ChIP grade protein G magnetic beads (#9006, CST) were added and incubated for 2 h at 4°C. The beads were sequentially rinsed with a low salt buffer, a high salt buffer, LiCl wash buffer, and TE buffer. Cross-linking was reversed with decrosslinking buffer (1% SDS, 0.1 M NaHCO3) overnight at 65°C. DNA fragments were purified using the MinElute PCR purification kit (#28004, Qiagen), and were analyzed by qPCR using SYBR green real time PCR (Bio-Rad). The ChIP efficiency of certain binding sites was evaluated using the percentage of chipped DNA against input chromatin. All ChIP primers are listed in the online supplemental table S5.

**RNA sequencing**

Total RNA was extracted from sh-NC (n=3) and *sh-TMPO-AS1* (n=3) PCa3 cells, and examined by RNA sequencing according to the manufacturer’s protocols. Genes with differential expression in sh-NC and *sh-TMPO-AS1* PCa3 cells were selected by using DEGseq according to a fold change of ≥ 1.5 and P ≤0.05. KEGG pathway enrichment analysis was performed for functional pathway analysis. RNA sequencing data were deposited at SRA (https://www.ncbi.nlm.nih.gov/sra/?term=) and are accessible via accession numbers [SRX8803980](https://www.ncbi.nlm.nih.gov/sra/SRX8803980%5baccn%5d).

**Bioinformatics analysis**

The genomic and clinical profiles of patients in the TCGA-PRAD dataset were analyzed in cBioPortal (3). The expressions of *TMPO-AS1* in primary PCa tissues and metastatic PCa tissues were examined based on the Gene Expression Omnibus (GEO) dataset GSE21034 (URL: https://www.ncbi.nlm.nih.gov/geo/query/acc.cgi?acc=GSE21034). The median of *TMPO-AS1* expression (TPM=1.123821557) was used as the cutoff value. Similarly, the cutoff value of *TMPO-AS1_L_* and *TMPO-AS1_S_* transcripts were defined as the median of expression value -1.2828 and -9.9658, respectively. Differentially expressed genes between the group of *TMPO-AS1*-H and *TMPO-AS1*-L were analyzed using the limma package in the R software with a P value threshold of 0.05 and 1.5-fold change. Based on the TCGA dataset and our RNA-seq data, differentially expressed genes were subjected to Gene Set Enrichment Analysis (GSEA) (<https://www.gsea-msigdb.org/gsea/index.jsp>) and Kyoto Encyclopedia of Genes and Genomes (KEGG) enrichment analysis (<https://david.ncifcrf.gov>/).

**Reference**

1. Li Q, Wang M, Hu Y, Zhao E, Li J, Ren L, et al. MYBL2 disrupts the Hippo-YAP pathway and confers castration resistance and metastatic potential in prostate cancer. Theranostics. 2021;11(12):5794-812.

2. Lang C, Yin C, Lin K, Li Y, Yang Q, Wu Z, et al. m(6) A modification of lncRNA PCAT6 promotes bone metastasis in prostate cancer through IGF2BP2-mediated IGF1R mRNA stabilization. Clin Transl Med. 2021;11(6):e426.

**
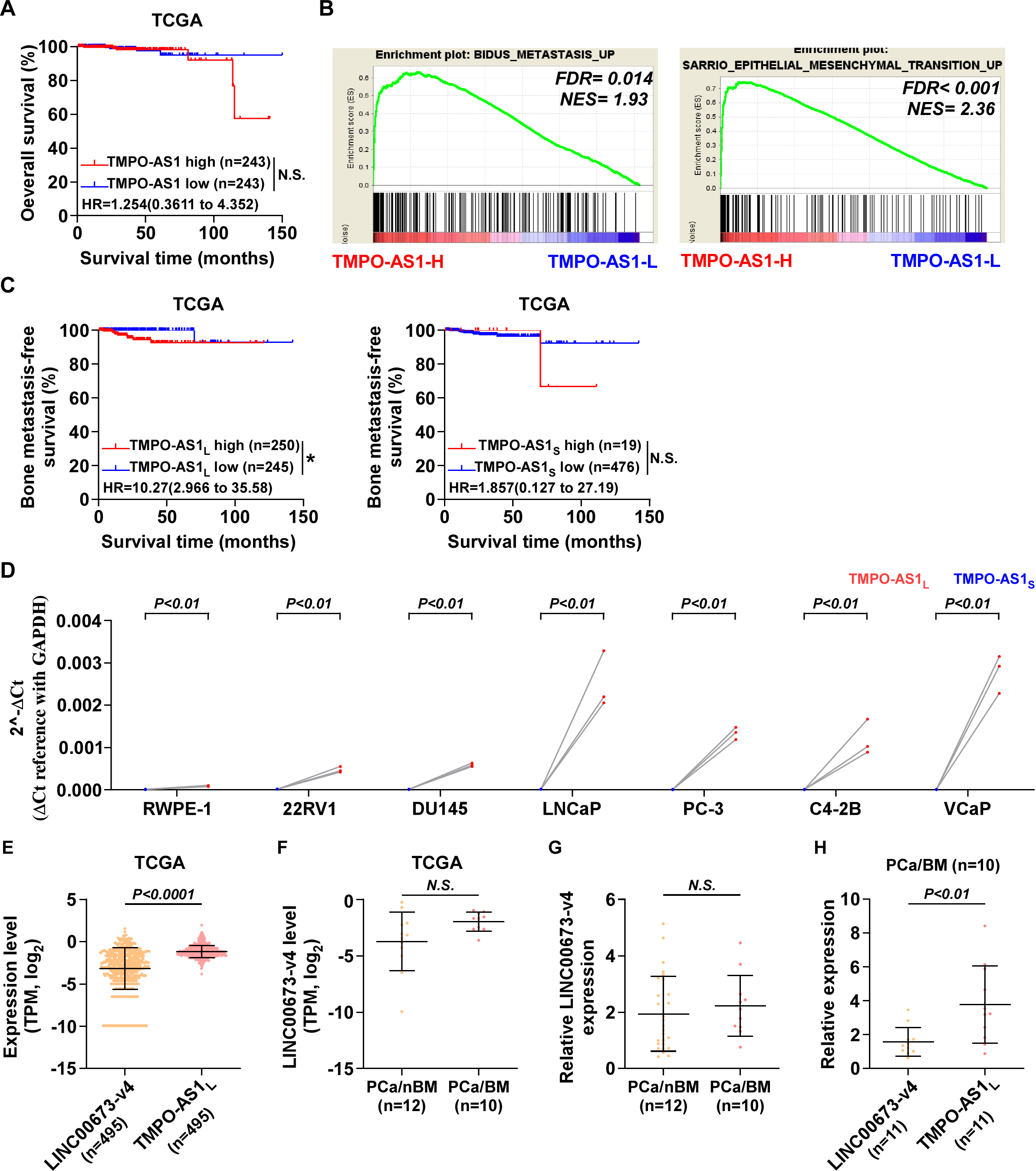
Supplementary Figure 1.** (A) Kaplan–Meier analysis of overall survival curves of the PCa patients stratified by *TMPO-AS1* expression in TCGA dataset. **P < 0.05*. *N.S. =* no significance. (B) Gene set enrichment analysis (GSEA) revealed that *TMPO-AS1* expression was correlated with metastasis and EMT signatures. (C) Kaplan–Meier analysis of bone metastasis‐free survival curves of the PCa patients stratified by *TMPO-AS1_L_* (left) and *TMPO-AS1_S_* (right) expression in TCGA dataset. **P < 0.05*. *N.S. =* no significance. (D) 2^-ΔCt value of *TMPO-AS1_L_* and *TMPO-AS1_S_* in normal prostate epithelial cell (RWPE-1) and PCa cell lines (DU145, LNCaP, PC-3, C4-2B and VCaP) by qRT‐PCR analysis relative to *GAPDH*. **P* < 0.05 by Wilcoxon matched-pairs signed rank test. (E) Comparison between expression of *LINC00673-v4* and *TMPO-AS1_L_* in the TCGA‐PRAD dataset. *P* value was determined by t test. (F) Comparison of *LINC00673-v4* expression between PCa/nBM and PCa/BM in TCGA dataset. *P* value was determined by Mann-Whitney test. *N.S. =* no significance. (G) qRT‐PCR analysis of *LINC00673-v4* in PCa/nBM (n = 27) and PCa/BM (n = 11). Transcript levels were normalized to *GAPDH*. *N.S. =* no significance. *P* value was determined by Mann-Whitney test. (H) qRT‐PCR analysis of *LINC00673-v4* and *TMPO-AS1_L_* in PCa/BM (n = 11). Transcript levels were normalized to *GAPDH*. *P* *< 0.05*. by Mann-Whitney test.

**
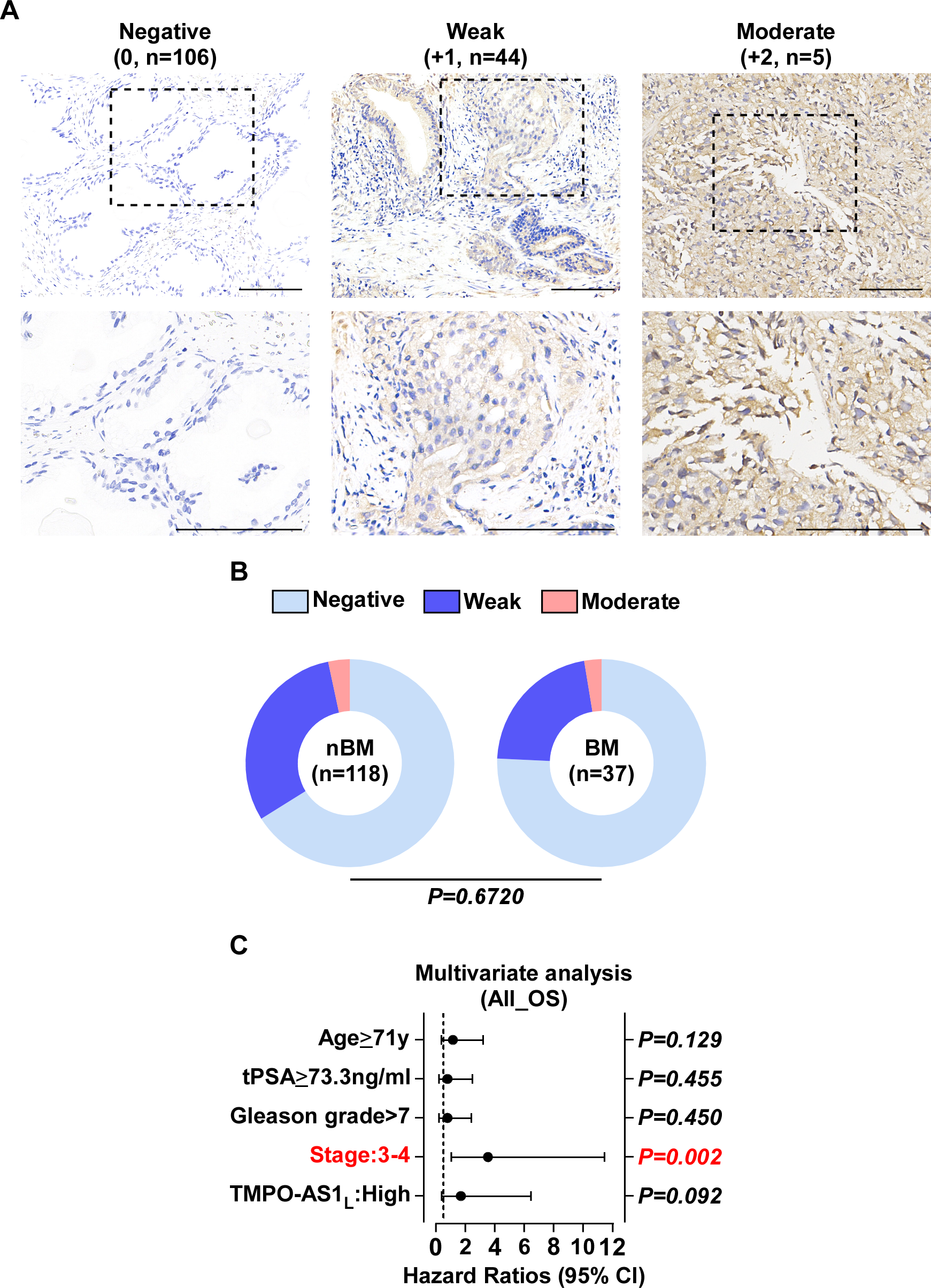
Supplementary Figure 2.** **No significant difference in *TMPO-AS1_S_* expression was found between PCa/nBM and PCa/BM.** (A) Representative images of *TMPO-AS1_S_* ISH staining in 118 PCa/nBM and 37 PCa/BM specimens, scored as negative (0), weak (+ 1) or moderate (+ 2). The scores are provided in brackets. Scale bar, 100μm. (B) ISH staining distribution for *TMPO-AS1_L_* in PCa/nBM and PCa/BM patient specimens. The P value was determined using as χ2 test. (C) The significance of the association between *TMPO-AS1_L_* signature and OS together other important clinical variables assessed using multivariate Cox regression analysis.

**
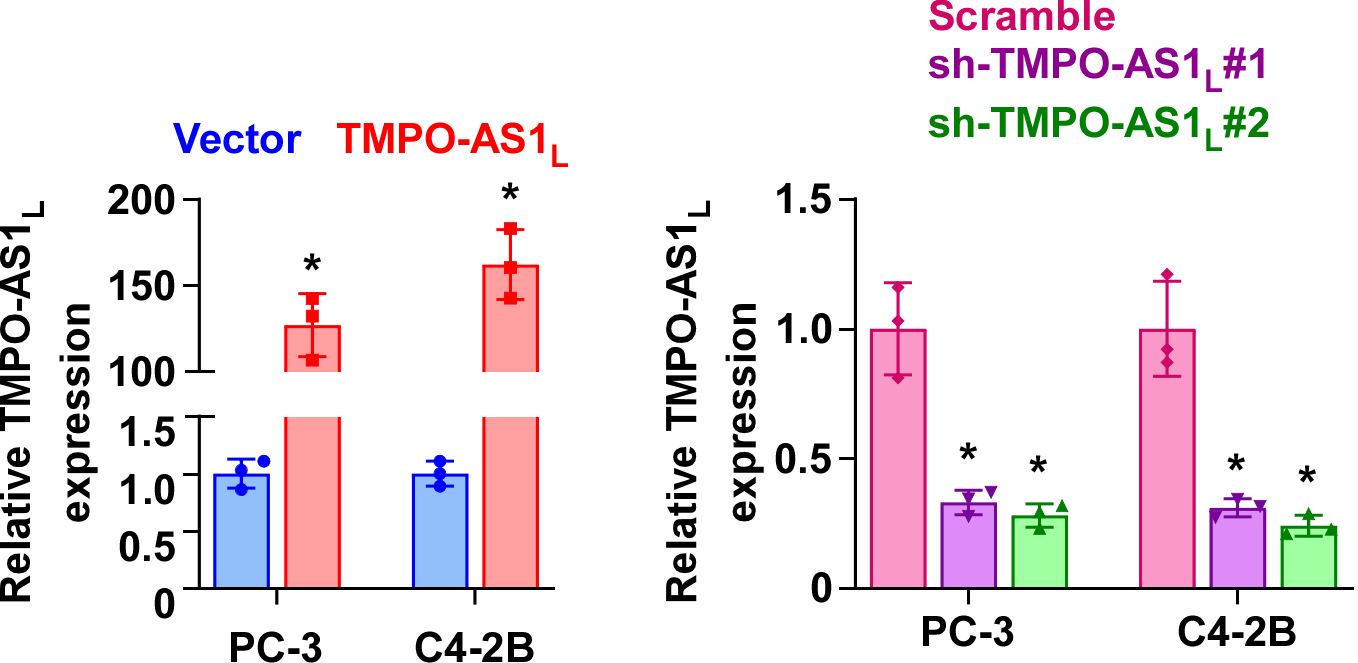
**

**Supplementary Figure 3.** Analysis of *TMPO-AS1_L_* expression in exogenously overexpressed *TMPO-AS1_L_* and *TMPO-AS1_L_*-stably downexpressing in bone metastatic PCa cell lines PC-3 and C4-2B. Each bar represents the mean values ± SD of three independent experiments. **P* < 0.05 by unpaired t test or one-way ANOVA test.

**
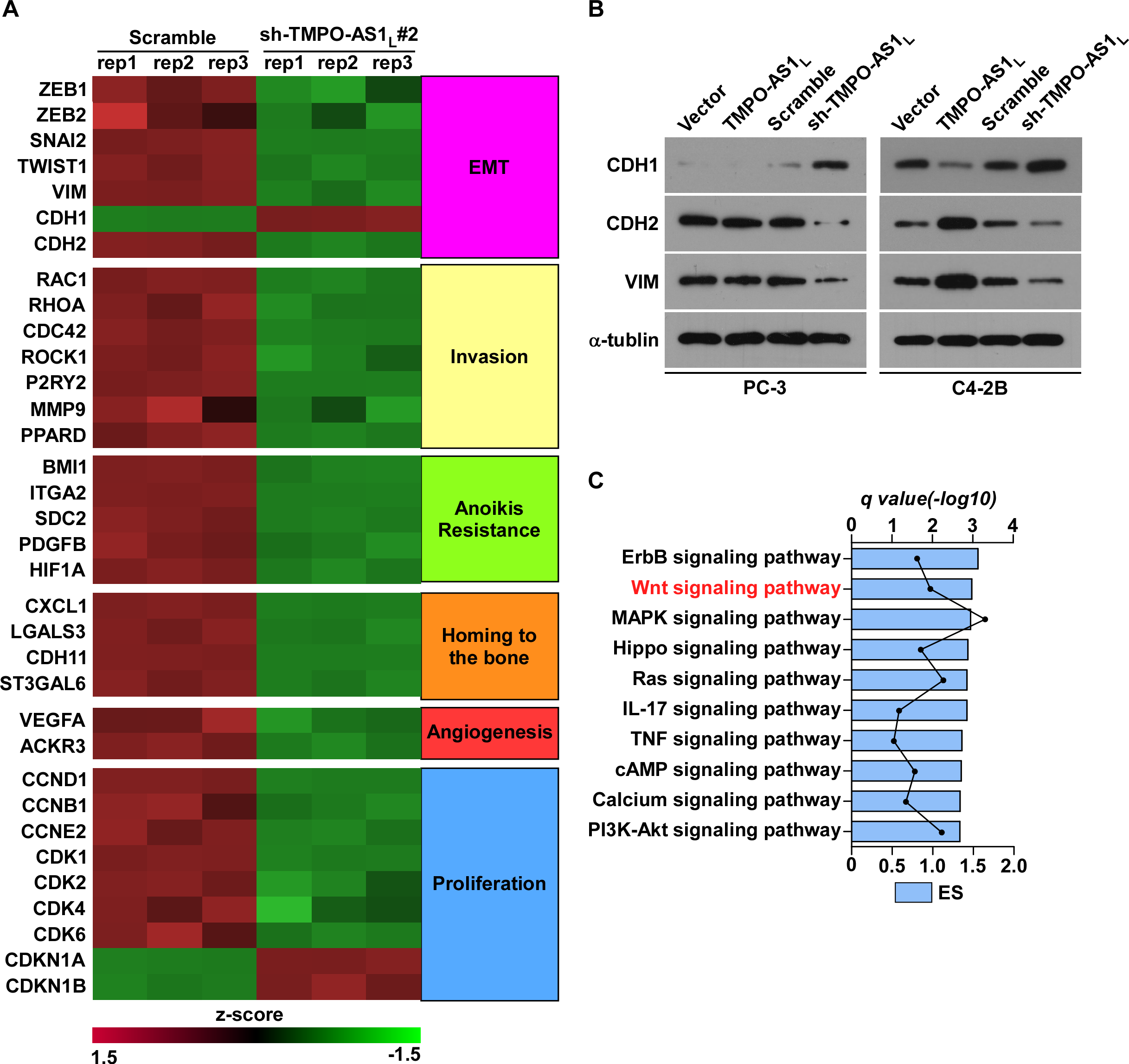
Supplementary Figure 4.** (A) Heatmap of the 34 *TMPO-AS1_L_*-regulated genes in the 6 clusters associated with bone metastasis. Pseudo-color scale values were log2 transformed. (B) Western blot analysis of epithelial marker, CDH1 (E-cadherin) and mesenchymal marker VIM (vimentin) and CDH2 (N-cadherin) in the indicated PCa cells. α-tubulin served as the loading control. (C) The top 10 signiﬁcant pathways enriched from the *TMPO-AS1_L_*-regulated genes in KEGG analysis.

**
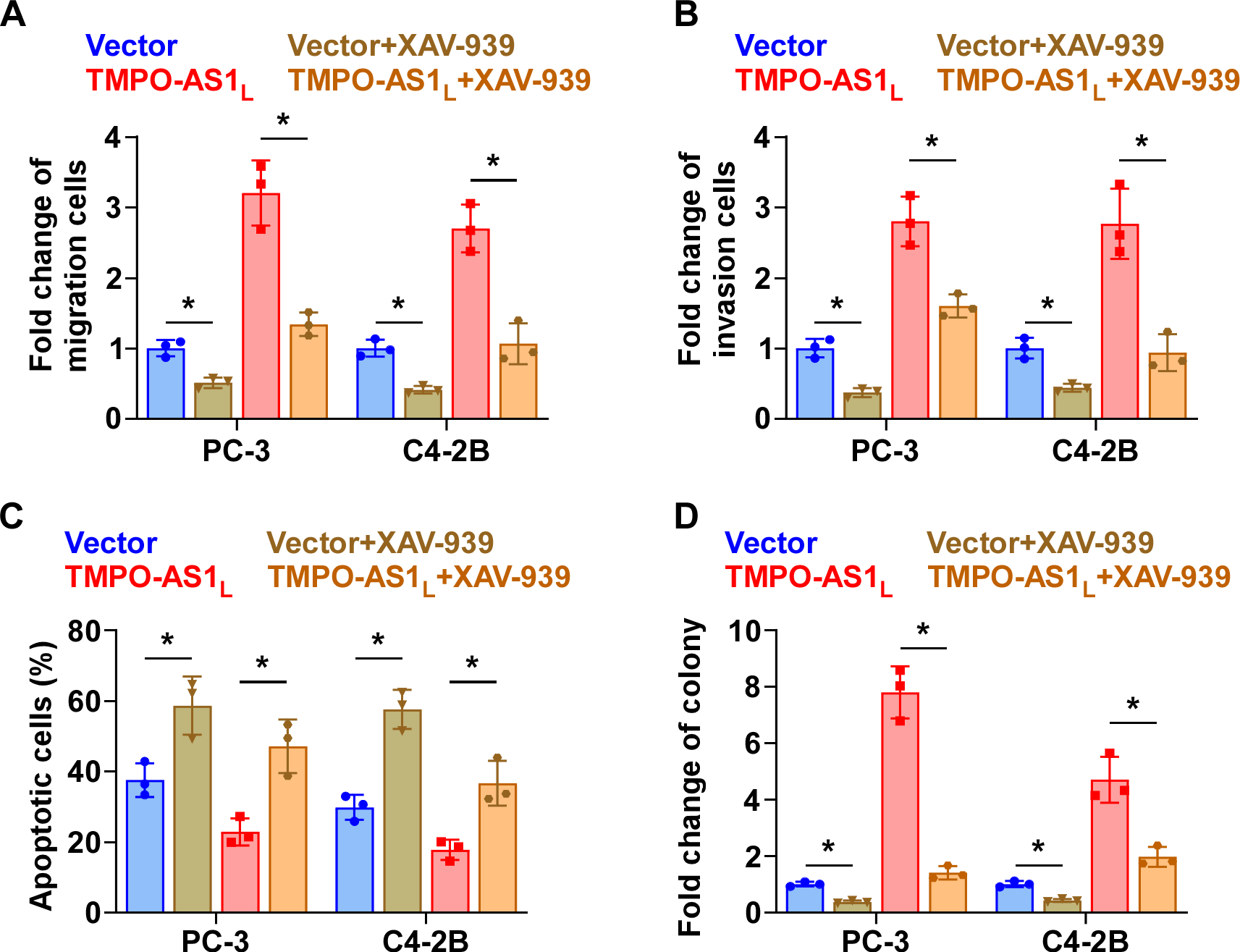
Supplementary Figure 5.** **The Wnt/β-catenin signaling is essential for biological functions of *TMPO-AS1_L_* in PCa cells *in vitro*.** Transwell assays showing migration (A) and invasion (B) ability of the indicated cells. Cell viability was evaluated by anoikis resistance assays (C) and cell proliferative potential was evaluated by colony formation assays (D) in the indicated cells. Each bar represents the mean values ± SD of three independent experiments. **P* <0.05 by one-way ANOVA test.

**
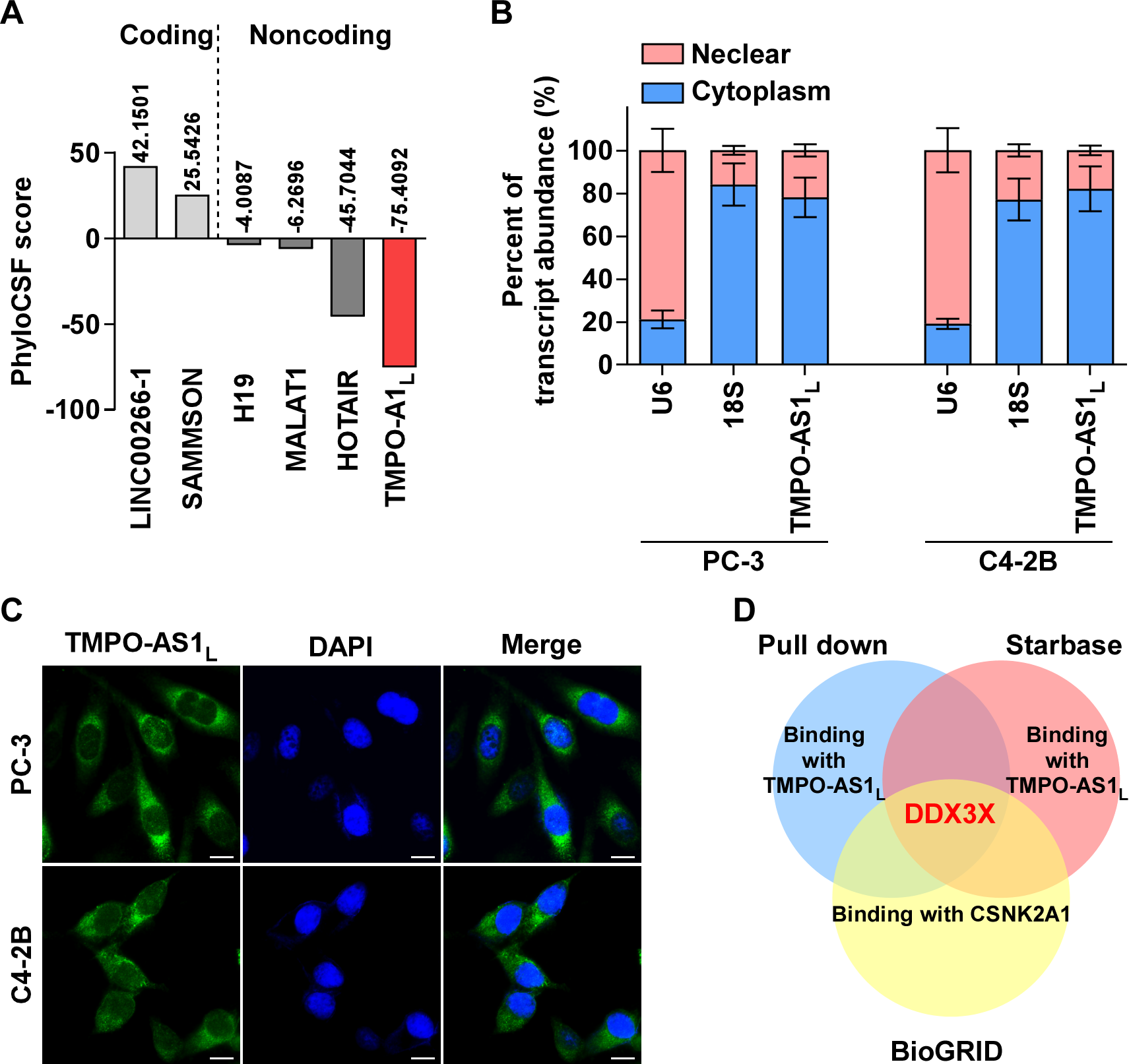
Supplementary Figure 6.** ***TMPO-AS1_L_* may interact with DDX3X in PCa.** (A) PhyloCSF analysis was performed to assess the maximum cerebrospinal fluid (CSF) scores of *TMPO-AS1_L_* and other known coding and noncoding lncRNAs. (B) Nuclear‐cytoplasmic fractionation assays revealing *TMPO-AS1_L_* expression in the cytoplasm and nucleus of PCa cells. U6 and 18S were used as positive controls in the nucleus and cytoplasm, respectively. Each bar represents the mean values ± SD of three independent experiments. **P* <0.05 by χ2 test. (C) RNA FISH showing the subcellular localization of *TMPO-AS1_L_* in PCa cells. Scale bar, 10μm. (D) Venn diagram of RBP which interacts with *TMPO-AS1_L_* in the result of MS or Starbase, and RBP which interacts with CSNK2A1 in BioGRID.

**
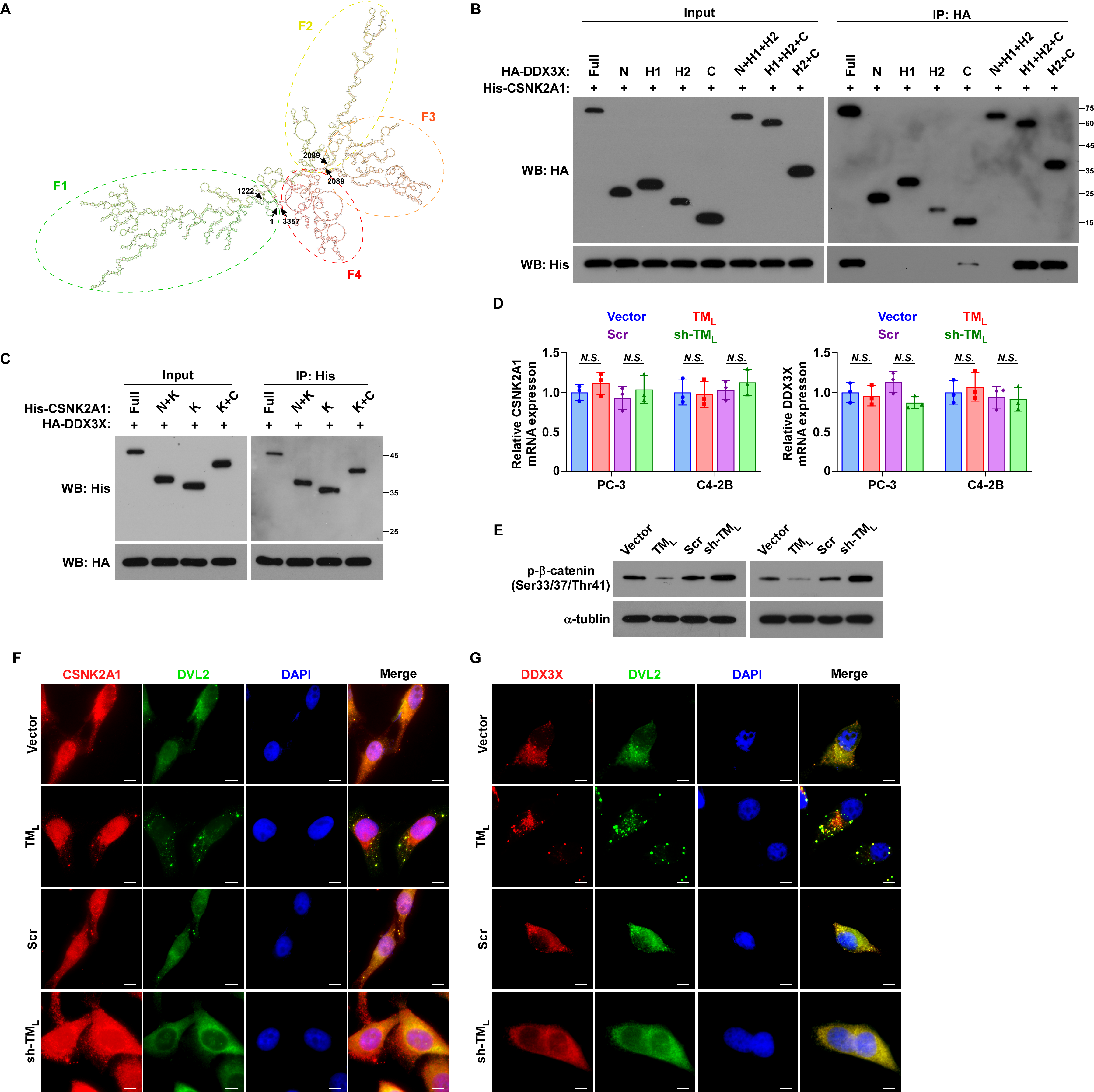
Supplementary Figure 7.** (A) Secondary structure of *TMPO-AS1_L_* predicted by RNAfold Website, which consists of four stem-loop structures. (B) 293 T cells were transfected with indicated HA-DDX3X truncations and GST-CSNK2A1, followed by IP assays with HA-beads to examine the domain of DDX3X interaction with CSNK2A1. (C) 293 T cells were transfected with indicated GST-CSNK2A1 truncations and HA-DDX3X, followed by IP assays with GST-beads to examine the domain of CSNK2A1 interaction with DDX3X. (D) qRT‐PCR analysis of CSNK2A1 (left) and DDX3X (right) expression in PCa cells of the indicated groups. Each bar represents the mean values ± SD of three independent experiments. *N.S.* = no significance. *P* value was determined by one-way ANOVA test. (E) Protein level of Phospho-β-Catenin (Ser33/37/Thr41) in PCa cells of the indicated groups. α-tubulin was used as loading controls. (F and G) Immunofluorescence staining for CSNK2A1, DDX3X and DVL2 in PC-3 cells of the indicated groups. Representative imagings showed that CSNK2A1 (F) or DDX3X (G) were colocalized with and DVL2 in the cytoplasm and existed as puncta. Scale bar, 10μm. All experiment was independently performed three times. V, vector; TM_L_, *TMPO-AS1_L_*; Scr, scramble; sh-TM_L_, sh*-TMPO-AS1_L_* #2.

**
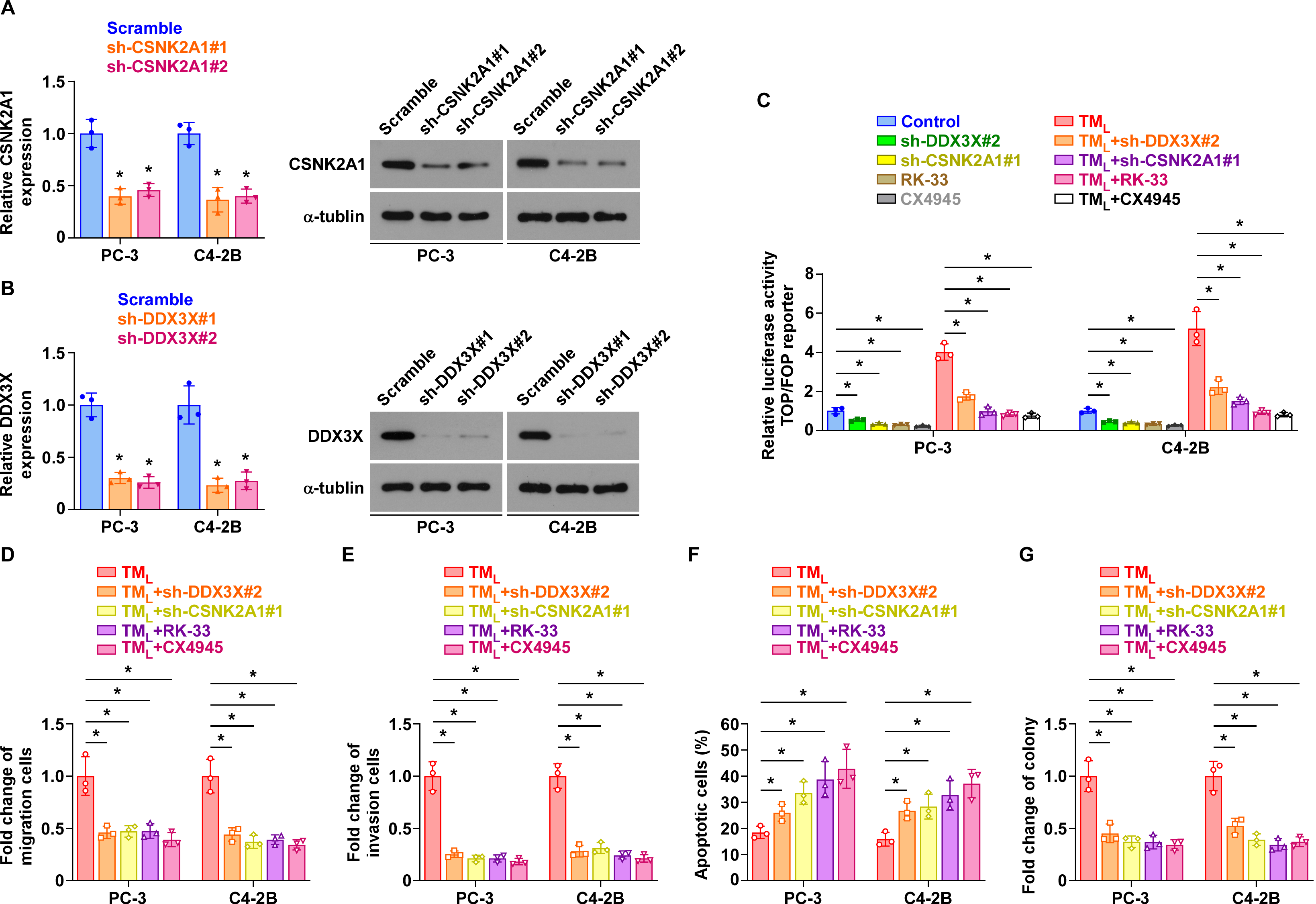
Supplementary Figure 8. *TMPO-AS1_L_* functions in a CSNK2A1/DDX3X dependent manner.** (A) qRT-PCR analysis of *CSNK2A1* expression in PCa cells of the indicated groups (left panel). Each bar represents the mean values ± SD of three independent experiments. **P* <0.05 by one-way ANOVA test. Transcript levels were normalized to *GAPDH* expression. Western blotting analysis of CSNK2A1 expression in the indicated cells. α-tubulin was used as loading controls (right panel). (B) qRT-PCR analysis of *DDX3X* expression in PCa cells of the indicated groups (left panel). Each bar represents the mean values ± SD of three independent experiments. **P* <0.05 by one-way ANOVA test. Transcript levels were normalized to *GAPDH* expression. Western blotting analysis of DDX3X expression in the indicated cells. α-tubulin was used as loading controls (right panel). (C) The TOP/FOP reporter activity in PCa cells of the indicated groups. Each bar represents the mean values ± SD of three independent experiments. **P* <0.05 by one-way ANOVA test. (D-G) Transwell assays showing migration (D) and invasion (E) ability of the indicated cells. Cell viability was evaluated by anoikis resistance assays (F) and cell proliferative potential was evaluated by colony formation assays (G) in the indicated cells. Each bar represents the mean values ± SD of three independent experiments. **P* <0.05 by one-way ANOVA test.

**
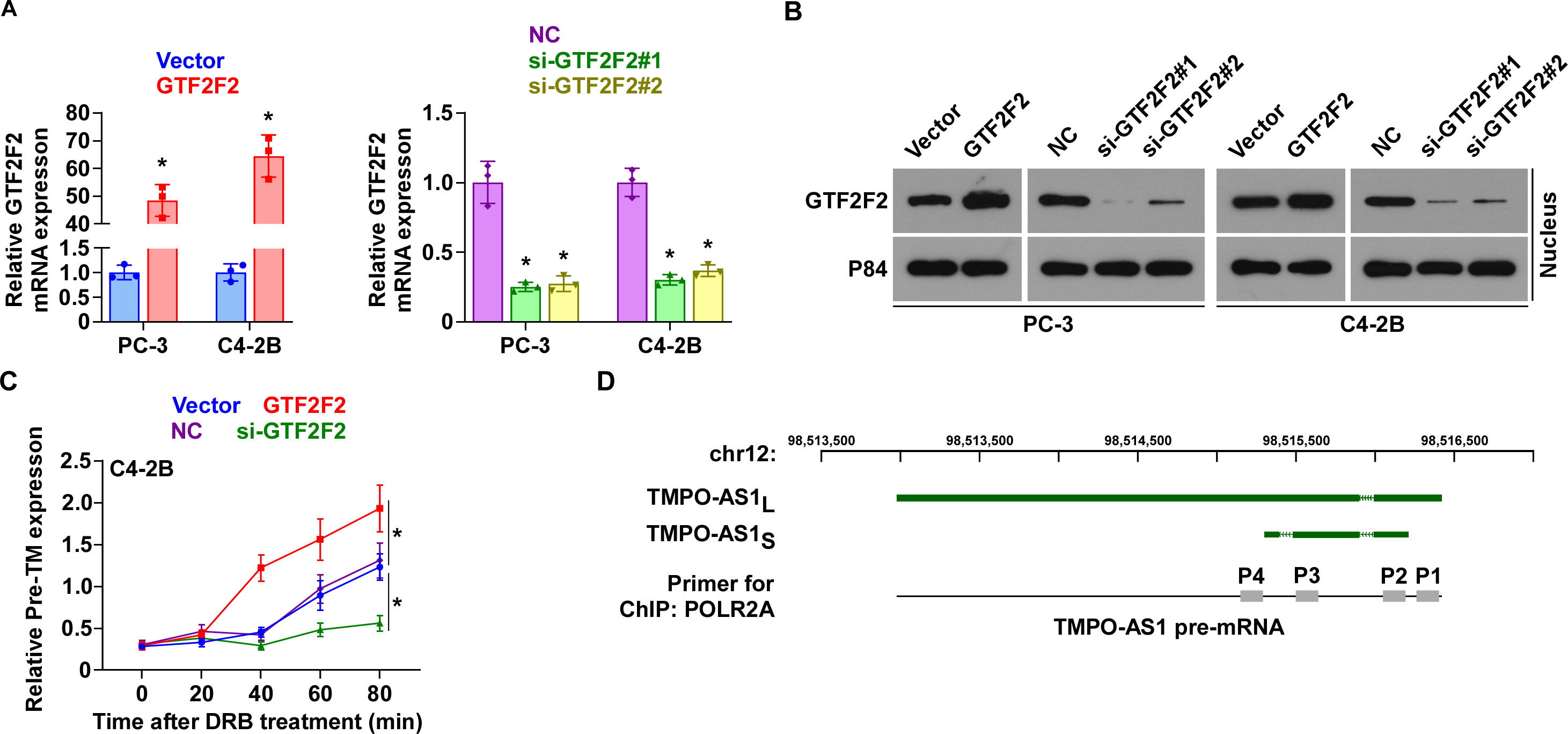
Supplementary Figure 9.** (A) qRT-PCR analysis of *GTF2F2* expression in PCa cells of the indicated groups. Each bar represents the mean values ± SD of three independent experiments. **P* <0.05 by t test or one-way ANOVA test. Transcript levels were normalized to *GAPDH* expression. (B) Protein levels of GTF2F2 expression in the nucleus were detected in PCa cells. P84 was used as loading controls. (C) C4-2B cells were treated after 48 h with DRB at 100 M for 3 h. DRB was removed, fresh medium was supplemented, and at the indicated times, cells were collected. Levels of *TMPO-AS1* pre-mRNA as assessed using qRT-PCR. Bars show the mean ± SD of three independently performed experiments. **P* < 0.05 according to repeated measures ANOVA. (D) Schematic representation of *TMPO-AS1* gene, drawn with exons (boxes) and introns (lines). The positions of the primers used to amplify RNA products by qRT-PCR are indicated (P1, the transcription start site of *TMPO-AS1_L_*; P2, the transcription start site of *TMPO-AS1_S_*; P3, before the splice site; and P4, distal region after splice site).

**Table S1. Clinicopathological features of 155 prostate cancer patients**

| Parameters | Number of cases |
| --- | --- |
| Age (years) |  |
| <71 | 77 |
| ≥71 | 78 |
| Median | 71 |
| Differentiation |  |
| Well/moderate | 66 |
| Poor | 89 |
| Serum PSA at diagnosis, µg/ml |  |
| <73.3 | 77 |
| ≥73.3 | 78 |
| Median | 73.3 |
| SD | 416.8 |
| Mean | 206.3 |
| Gleason grade |  |
| ≤7 | 71 |
| >7 | 84 |
| *TMPO-AS1_L_* expression |  |
| High | 89 |
| Low | 66 |
| *TMPO-AS1_S_* expression |  |
| High | 5 |
| Low | 150 |
| BM status |  |
| nBM | 118 |
| BM | 37 |

**Abbreviation: PSA, Prostate-specific Antigen; SD, Standard Deviation; BM, Bone Metastasis; n-BM: Non-bone Metastasis.**

**Table S2. Relationship between *TMPO-AS1_L_* and clinicopathological features in 155 patients with prostate cancer.**

| Parameters | Number of cases | *TMPO-AS1* expression | | *P*-values |
| --- | --- | --- | --- | --- |
|  |  | High | Low |  |
| Age (years) |  |  |  |  |
| <71 | 77 | 48 | 29 |  |
| ≥71 | 78 | 41 | 37 | 0.2185 |
| Differentiation |  |  |  |  |
| Well/moderate | 66 | 28 | 38 |  |
| Poor | 89 | 61 | 28 | 0.0011* |
| Serum PSA |  |  |  |  |
| <73.3 | 77 | 34 | 43 |  |
| ≥73.3 | 78 | 55 | 23 | <0.001* |
| Gleason grade |  |  |  |  |
| ≤7 | 71 | 31 | 40 |  |
| >7 | 84 | 58 | 26 | 0.0014* |
| BM status |  |  |  |  |
| nBM | 118 | 60 | 58 |  |
| BM | 37 | 29 | 8 | 0.0031* |

**Abbreviation: PSA, Prostate-specific Antigen; SD, Standard Deviation; BM, Bone Metastasis; n-BM: Non-bone Metastasis.**

**Table S3. List of *TMPO-AS1_L_* interacting proteins which is associated with Wnt pathway identified by mass spectrometry**

| Protein | Gene name | Unique  peptides | Area |
| --- | --- | --- | --- |
| P68400/CSK21_HUMAN | CSNK2A1 | 16 | 4.60×10^8^ |
| P15407/FOSL1_HUMAN | FOSL1 | 1 | 5.56×10^7^ |
| Q9HCK8/CHD8_HUMAN | CHD8 | 17 | 5.42×10^7^ |
| P48729/KC1A_HUMAN | CSNK1A1 | 9 | 4.81×10^7^ |
| Q13555/KCC2G_HUMAN | CAMK2G | 9 | 3.78×10^7^ |
| Q9Y265/RUVB1_HUMAN | RUVBL1 | 9 | 2.51×10^7^ |
| Q01970/PLCB3_HUMAN | PLCB3 | 5 | 1.07×10^7^ |
| P05412/JUN_HUMAN | JUN | 3 | 6.49×10^6^ |
| Q15147/PLCB4_HUMAN | PLCB4 | 3 | 4.47×10^6^ |
| O94907/DKK1_HUMAN | DKK1 | 1 | 2.58×10^6^ |
| P17612/KAPCA_HUMAN | PRKACA | 1 | 1.97×10^6^ |
| P49674/KC1E_HUMAN | CSNK1E | 1 | 1.96×10^6^ |
| Q92793/CBP_HUMAN | CREBBP | 1 | 1.04×10^6^ |
| P22694/KAPCB_HUMAN | PRKACB | 1 | 6.04×10^5^ |
| Q09472/EP300_HUMAN | EP300 | 1 | 3.44×10^5^ |
| Q13557/KCC2D_HUMAN | CAMK2D | 1 | 4.59×10^2^ |

**Table S4. List of primers used for quantitative real-time RT-PCR.**

| **Primer** | |
| --- | --- |
| *TMPO-AS1_L_*-F | AGCAAGTTGTTAGGTAGGTT |
| *TMPO-AS1_L_*-R | GGCAGGAAGGAGAGTAGAA |
| *TMPO-AS1_S_*-F | ATCGGCGTCTCCTCTCGCTC |
| *TMPO-AS1s*-R | AACTGCGGCCAAGCTGGAAG |
| *GAPDH*-F | GTCTCCTCTGACTTCAACAGCG |
| *GAPDH*-R | ACCACCCTGTTGCTGTAGCCAA |
| *DDX3X*-F | ACTATGCCTCCAAAGGGTGTCC |
| *DDX3X*-R | AGAGCCAACTCTTCCTACAGCC |
| *GTF2F2*-F | GAAACTGCGGATTGCCAAGACTC |
| *GTF2F2*-R | ATGTCTGTCCTCCA ACACTTTGC |
| *CSNK2A1*-F | GGTGAGGATAGCCAAGGTTCTG |
| *CSNK2A1*-R | TCACTGTGGACAAAGCGTTCCC |
| *U6*-F | CTCGCTTCGGCAGCACAT |
| *U6*-R | TTTGCGTGTCATCCTTGCG |
| *18S*-F | ACCCGTTGAACCCCATTCGTGA |
| *18S*-R | GCCTCACTAAACCATCCAATCGG |
| Pre-*TMPO-AS1*-F | GCAGGTAGAGCTGGACGTACAC |
| Pre-*TMPO-AS1*-R | GAAGACCCCTCGGTCCTGACAAAAG |
| *LINC00673-V4*-F | CAAGCTGGAGGTGGAATCAGAGG |
| *LINC00673-V4*-R | GTCCTTCCCATCCTCTTTCTTG |

**Supplementary Table 5. List of primers used for ChIP assay**

| **Primer** | |
| --- | --- |
| P1-F | AACAACCGCCGCCAACGCAACTC |
| P1-R | CTGCAGGCGCCGGAGCGGAGAGG |
| P2-F | CAAACGCCCGCCTTTGTAGC |
| P2-R | TGACGAAGAGCGCGAGCCC |
| P3-F | AGGAGGCGAGCAGCCTGCTTC |
| P3-R | AGCGCGGCGGCTAATGGAAC |
| P4-F | GAATTCCACGCCAGTGTGAGGAC |
| P4-R | TATGAGTGCCTGCAGACAAGCCTCGCTTC |
