# Supplementary material for: The long transcript of lncRNA TMPO-AS1 promotes bone metastases of prostate cancer by regulating the CSNK2A1/DDX3X complex in Wnt/β-catenin signaling
Source: Cell Death Discov. 2023 Aug 5;9:287. doi: 10.1038/s41420-023-01585-w (PMC10403548; doi:10.1038/s41420-023-01585-w)

**Figure 5**

**B**

β-catenin (nucleus)

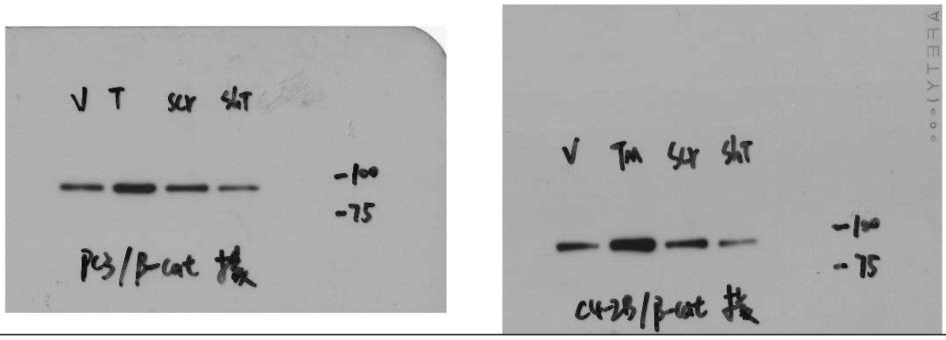


P84

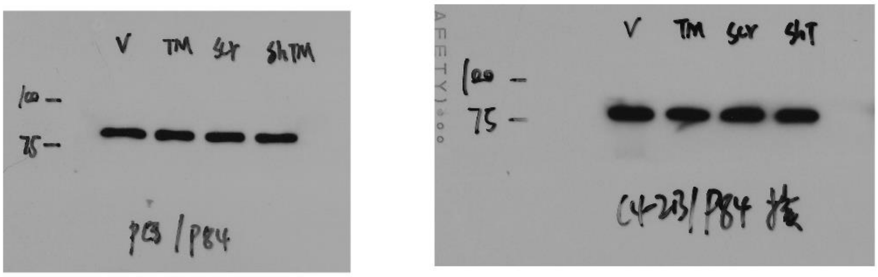


β-catenin (Total)

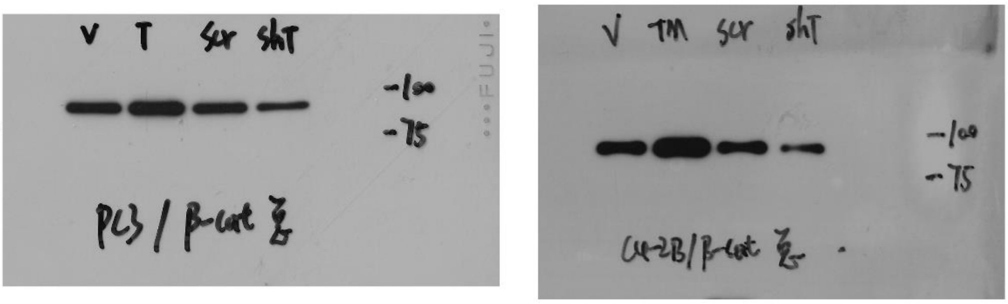


α-tublin

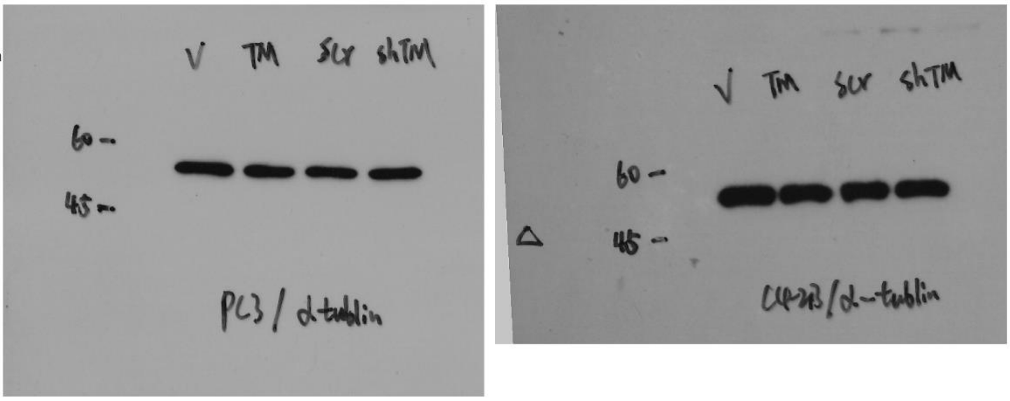

**Figure 5D**

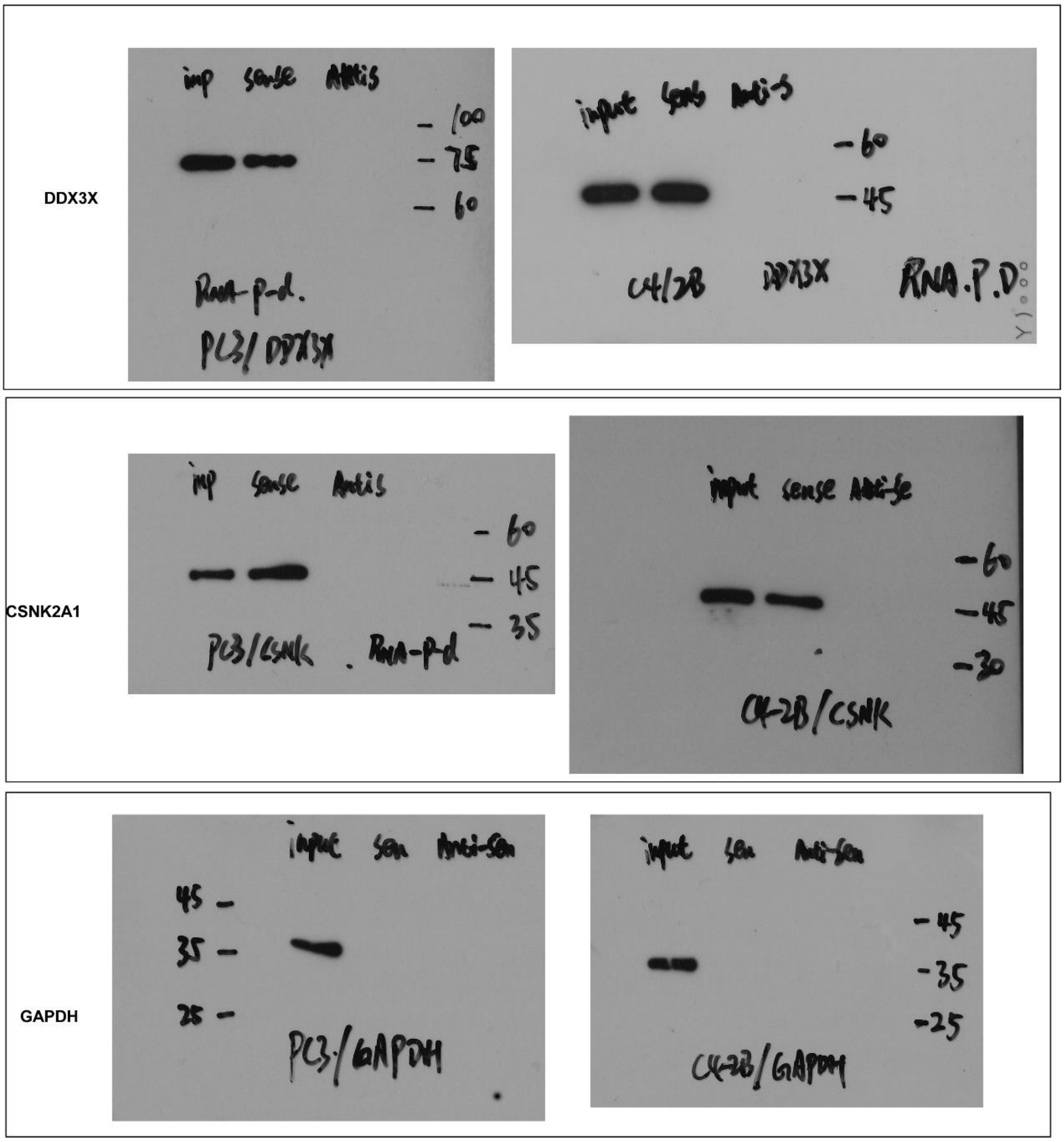
DDX3X
CSNK2A1
GAPDH
inp sense Antis
input sense Anti-S
PC3/DDX3X
C4/2B DDX3X RNA.P.D
PC3/CSNK RNA-P-d
input sense Anti-se
C4-2B/CSNK
input sen Anti-sen
PC3/GAPDH
C4-2B/GAPDH
100
75
60
45
35
30
25

**Figure 5F**

Streptavidin-HRP

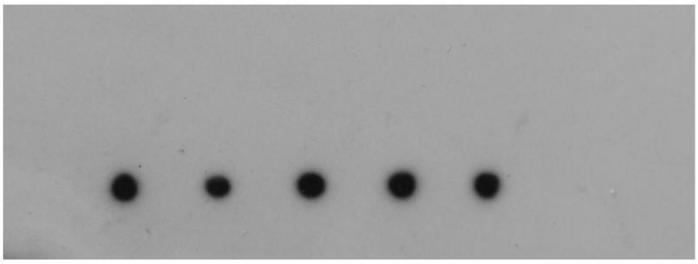

CSNK2A1

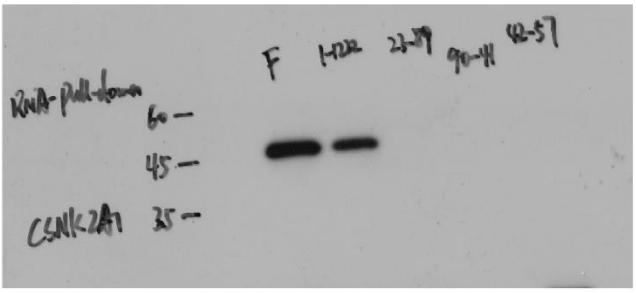

Input: CSNK2A1

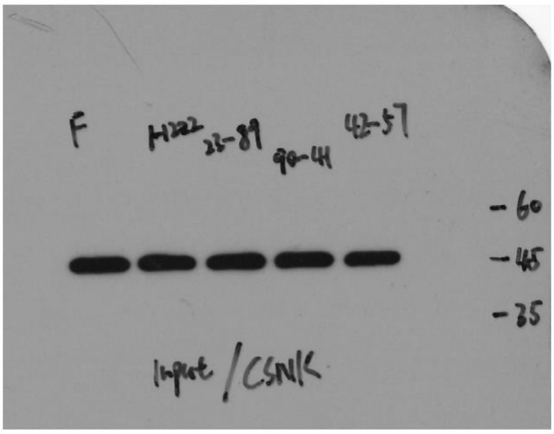

DDX3X

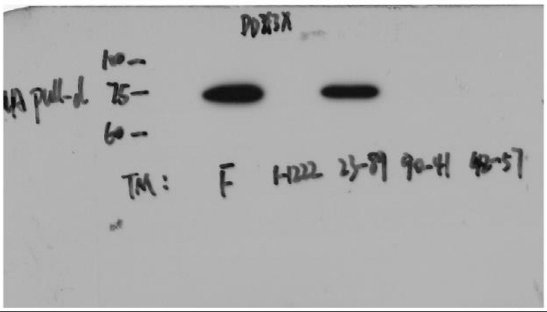

Input: DD3X

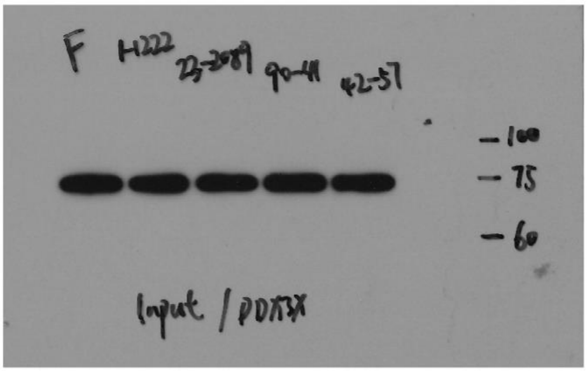

Figure 5G

CSNK2A1

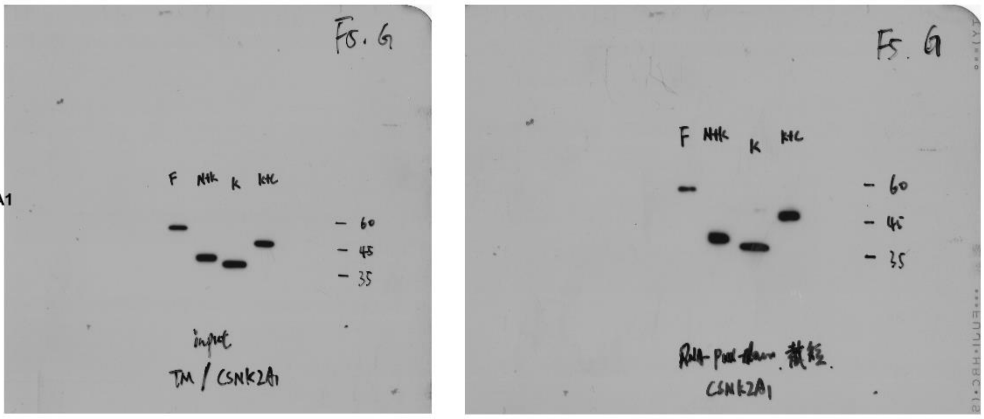


DDX3X

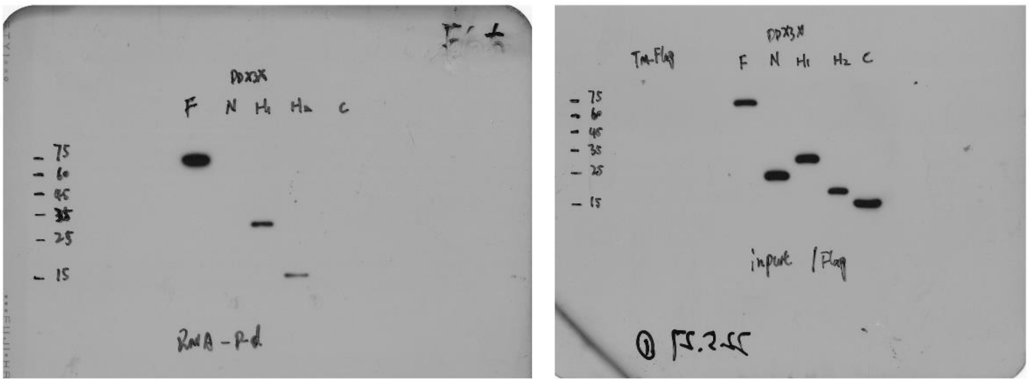

**Figure 5H**

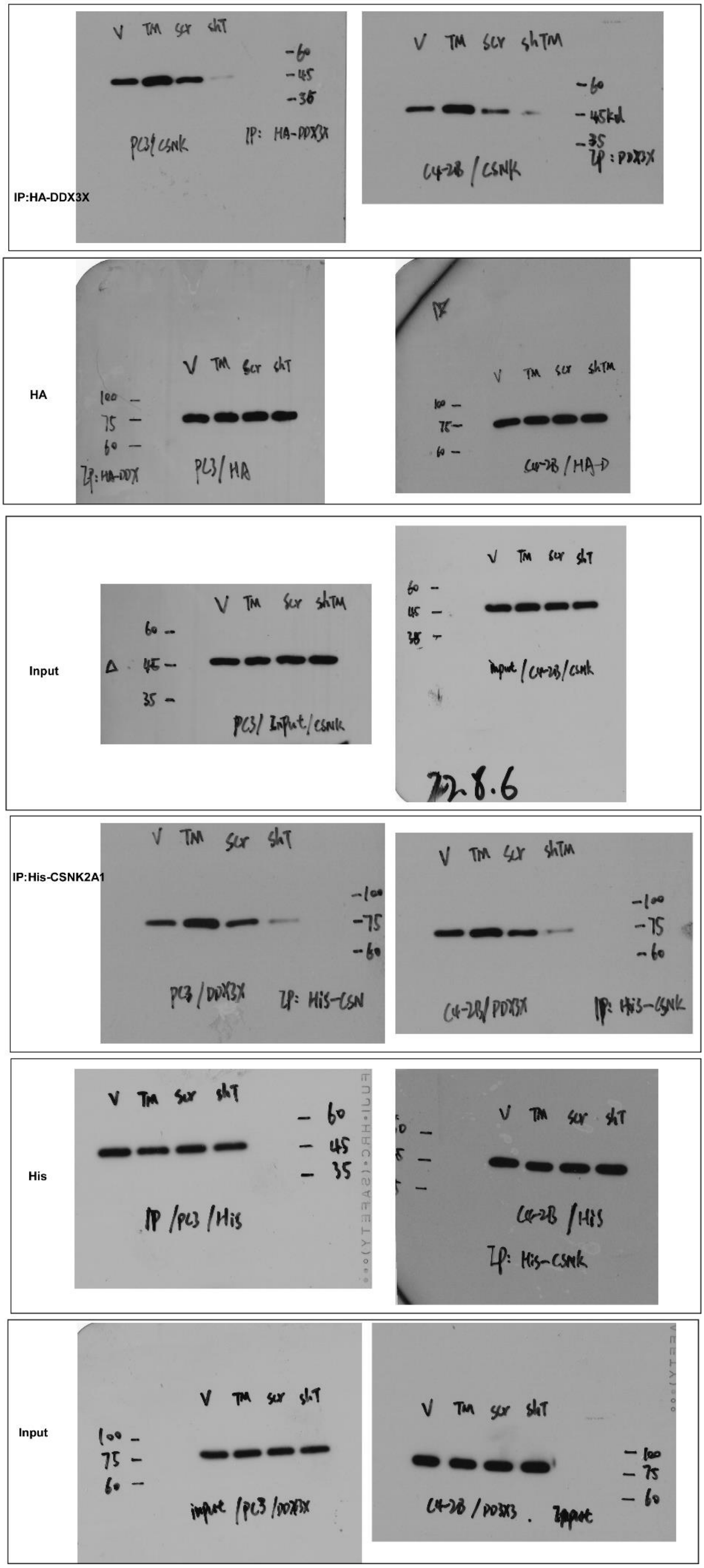

Figure 5I

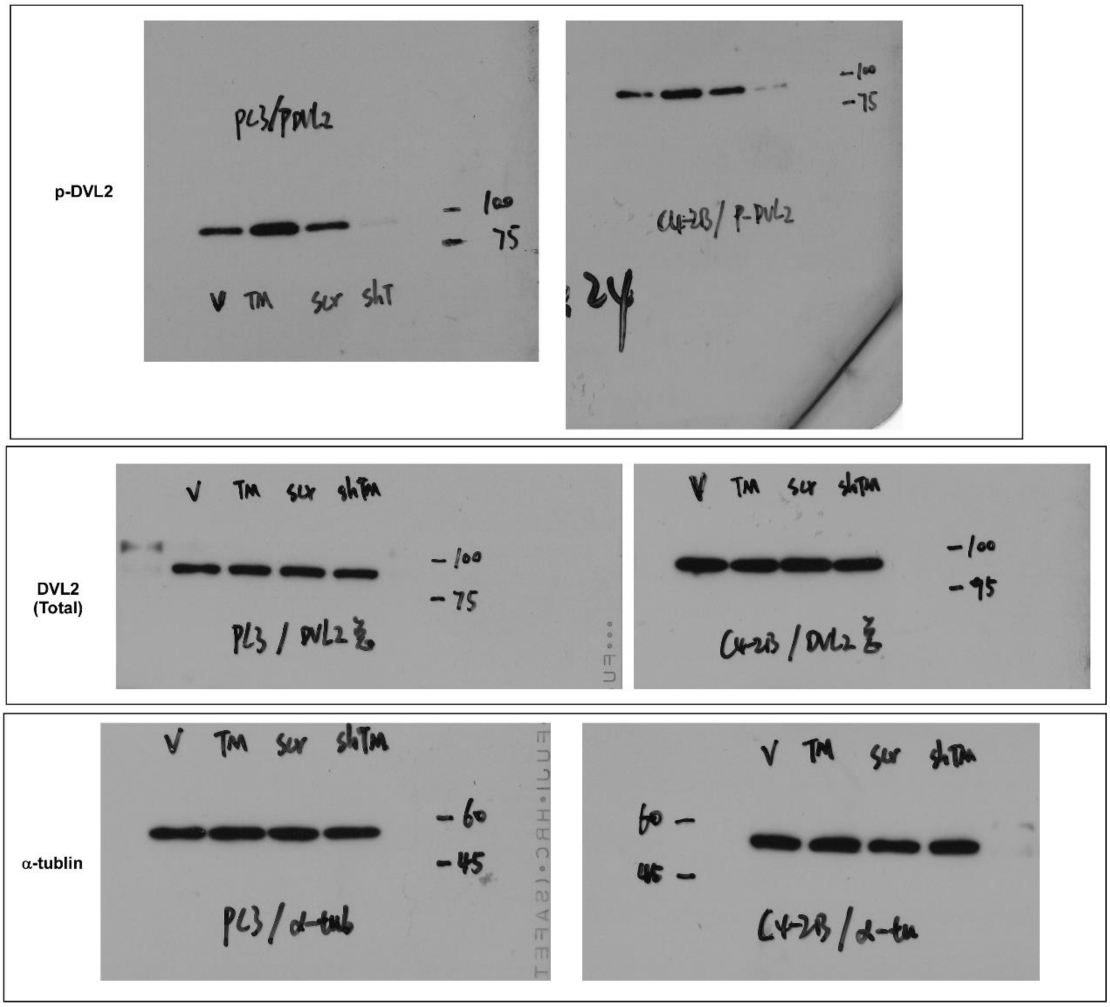

**Figure 6A**

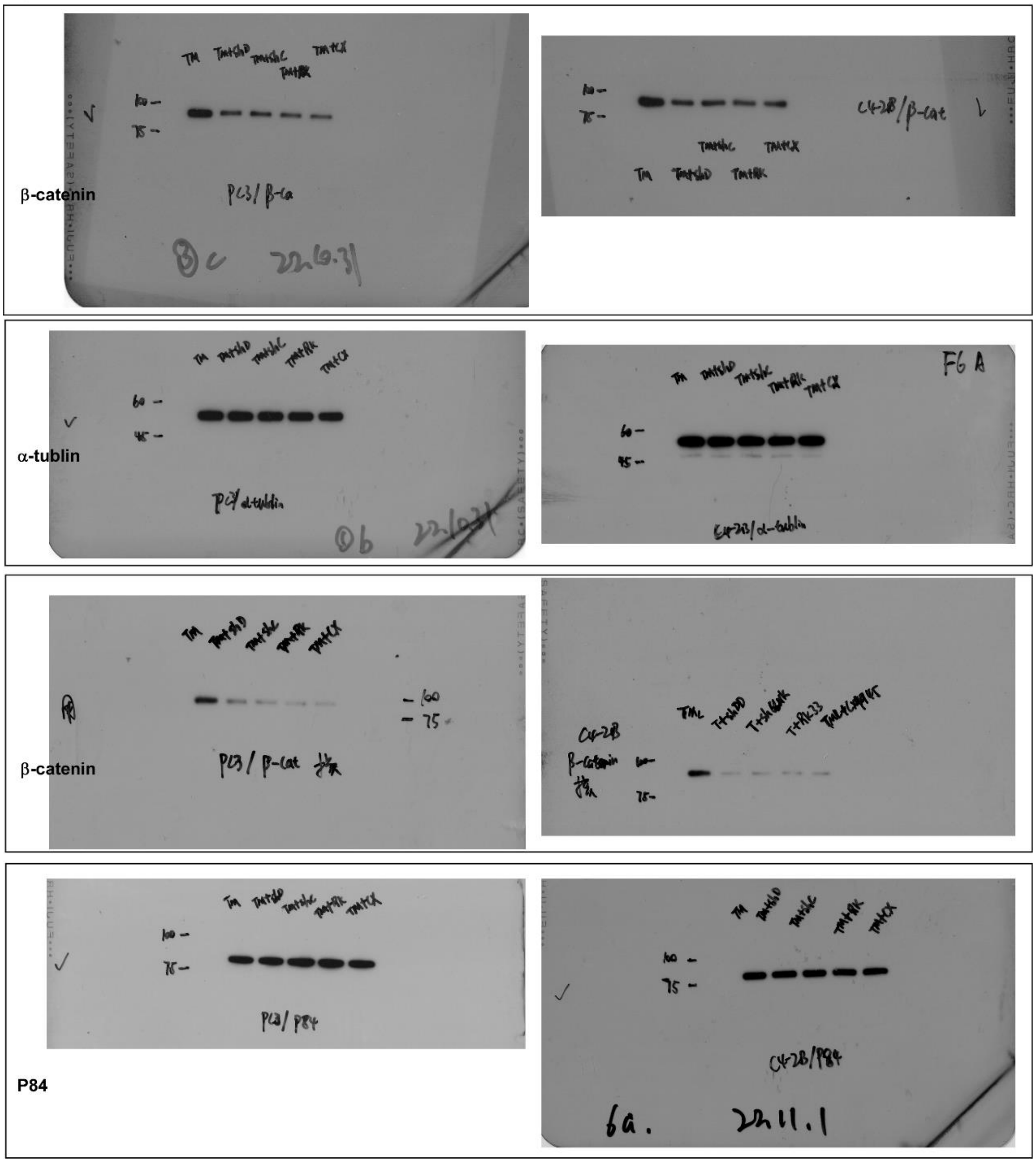
β-catenin
α-tublin
β-catenin
P84
C4-2B/β-cat
F6 A
PC3/P84
C4-2B/P84
6a. 22.11.1

**Figure 6A**

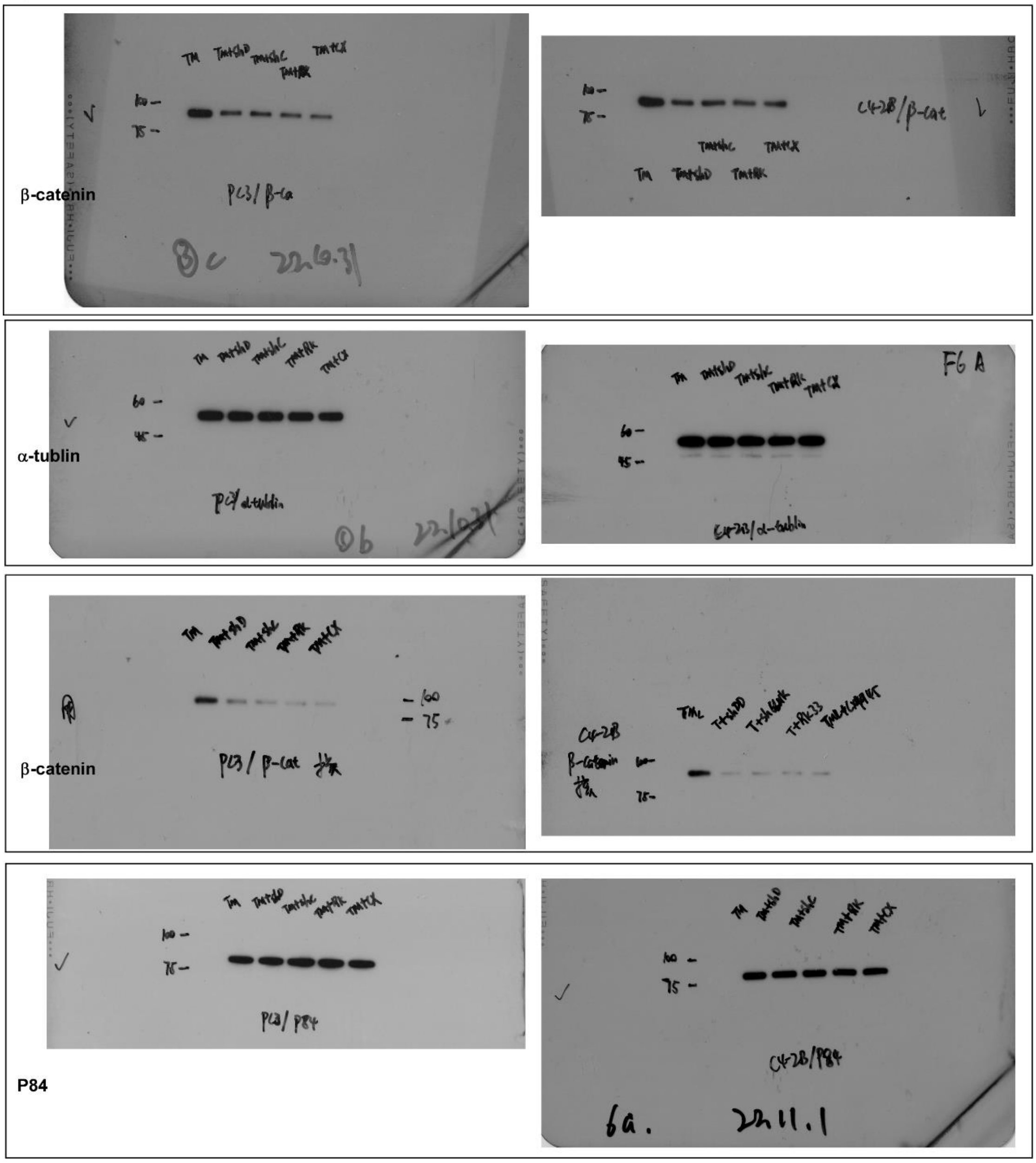

**Figure 6B**

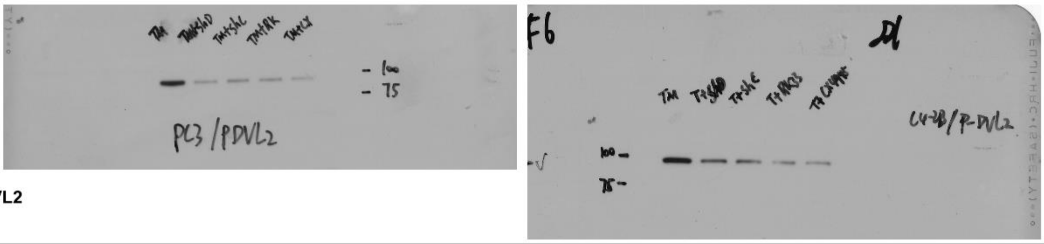


p-DVL2

DVL2 (Total)

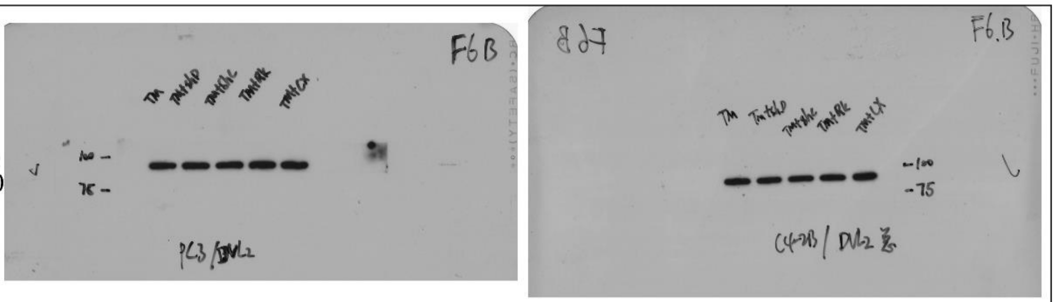


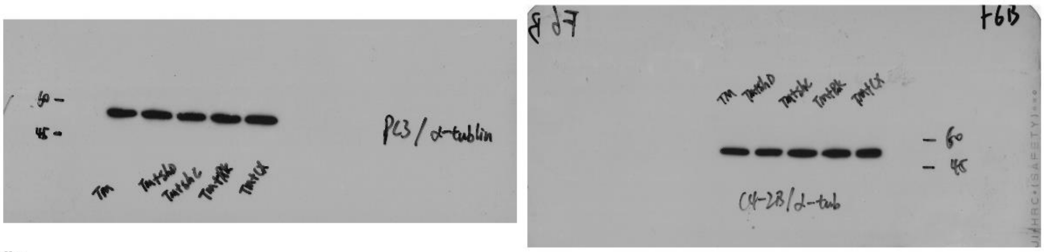


α-tublin

**Supplemental Figure 4**

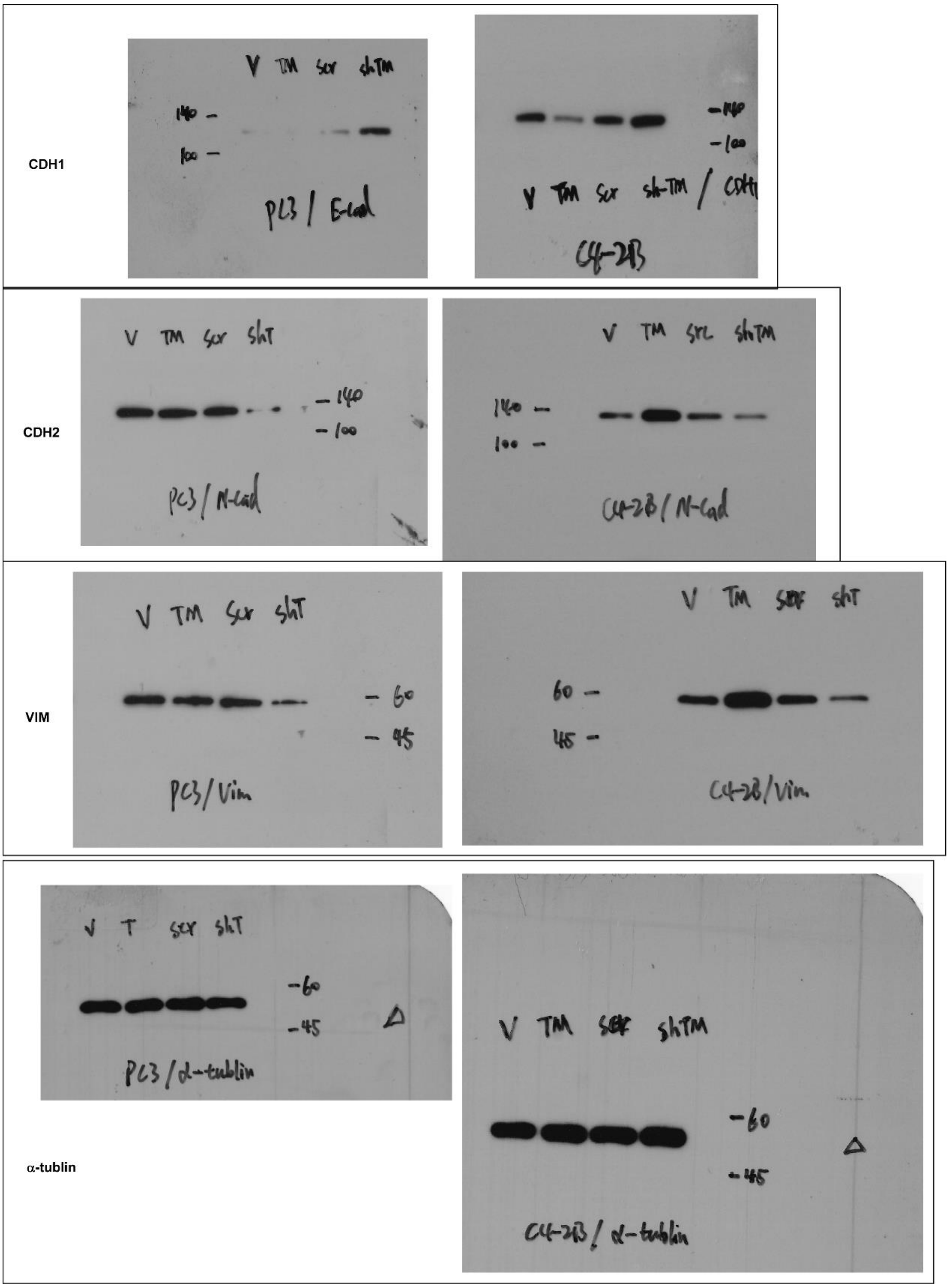

## Supplemental Figure 7B

WB: HA

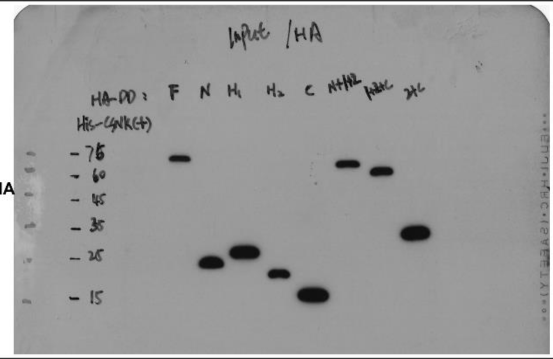

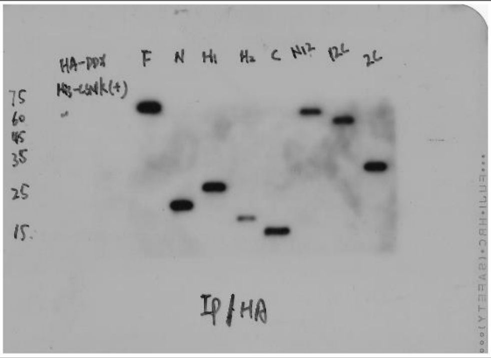

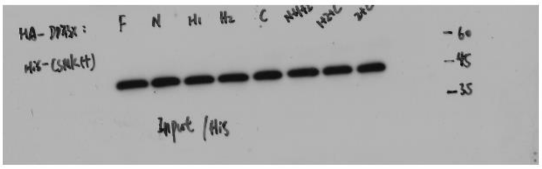

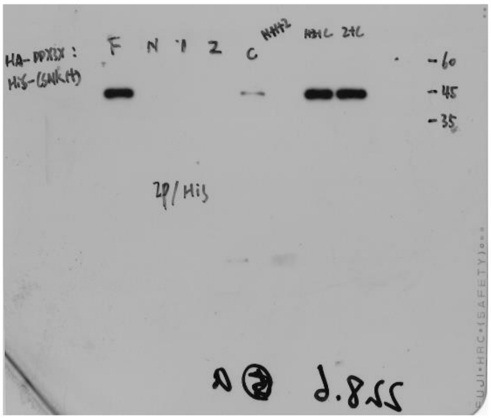

WB: His

## Supplemental Figure 7C

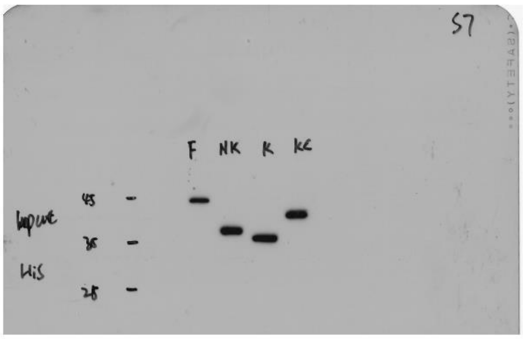

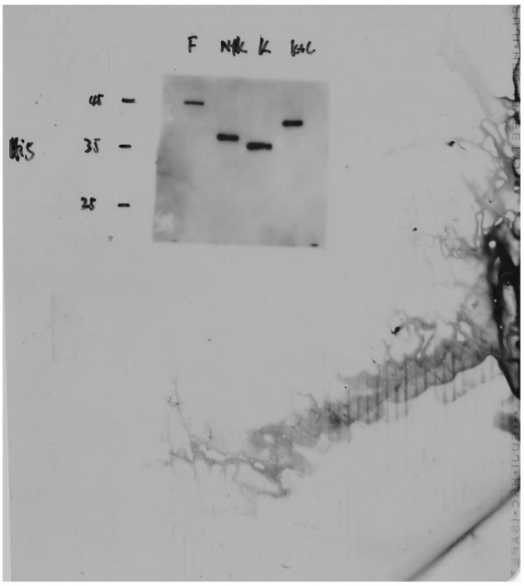

WB: His

WB: HA

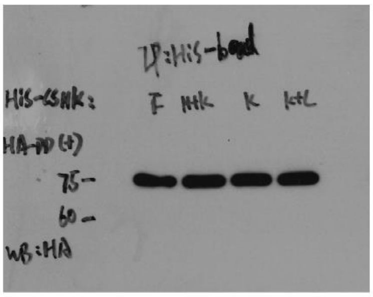

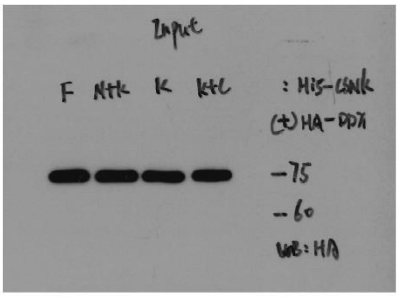

**Supplemental Figure 7E**

p-β-catenin (Ser33/37/Thr41)

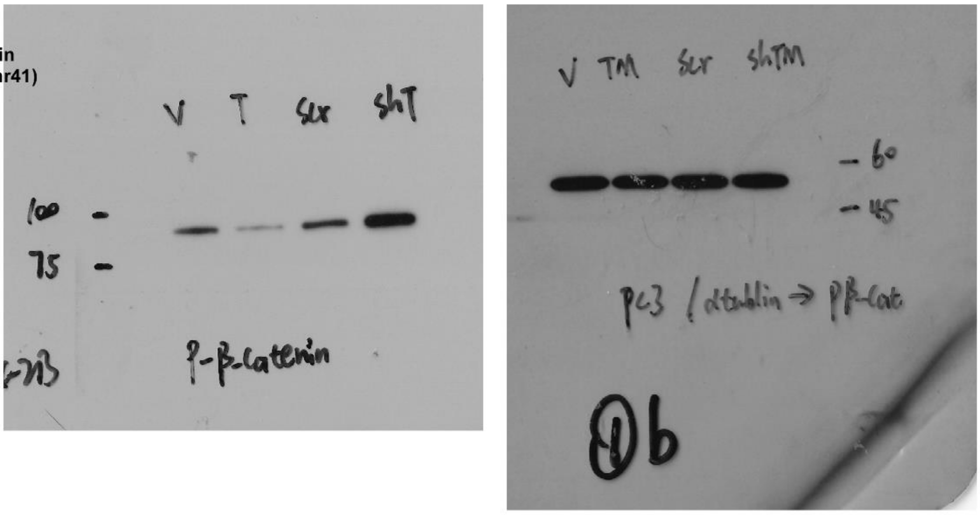


α-tublin

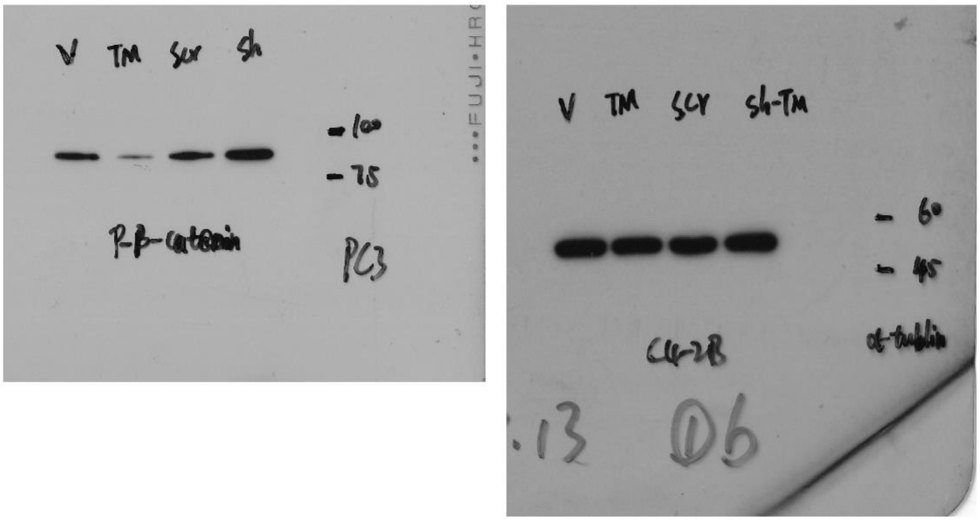

**Supplemental Figure 8**

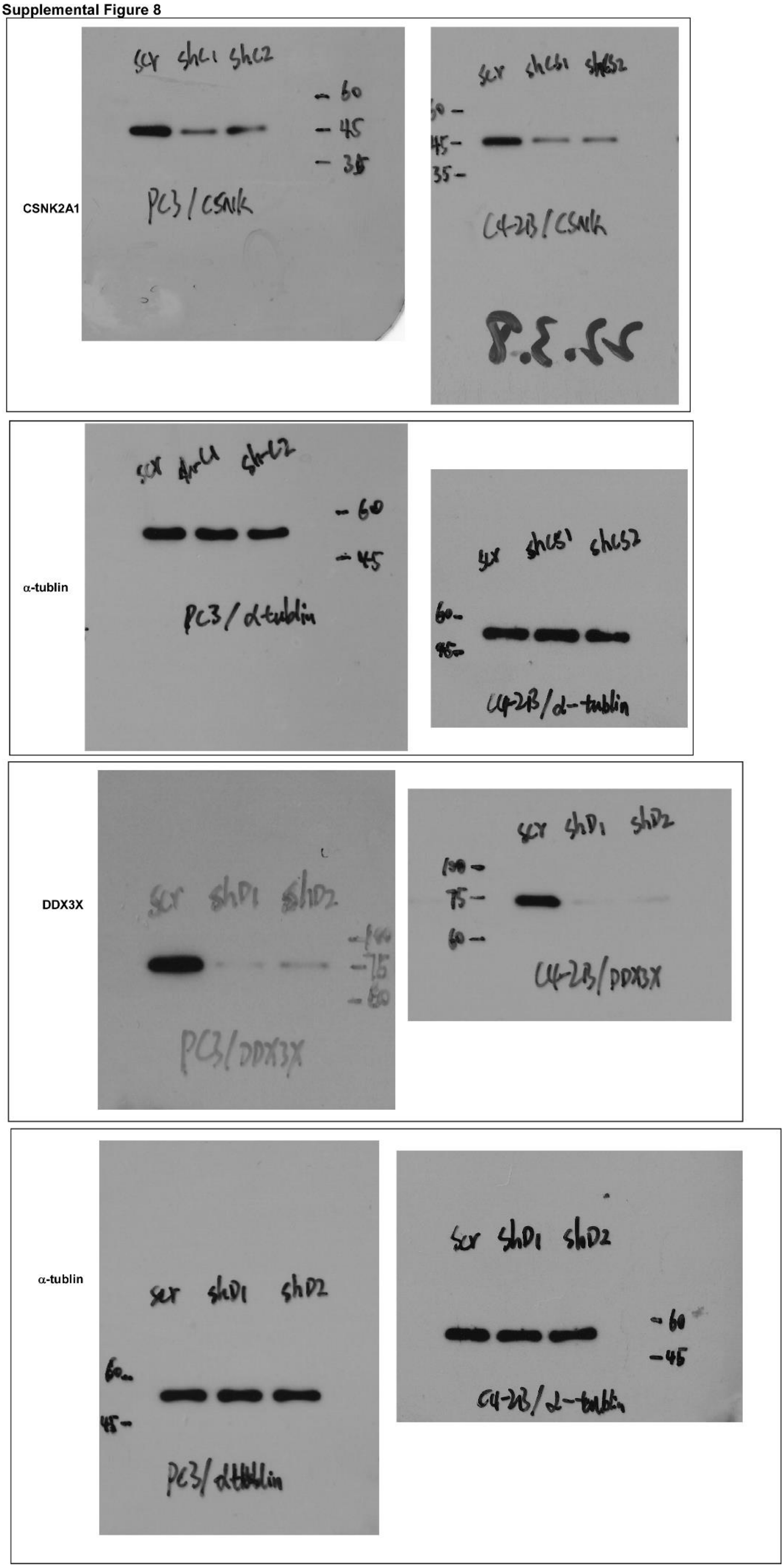

**Supplemental Figure 9**

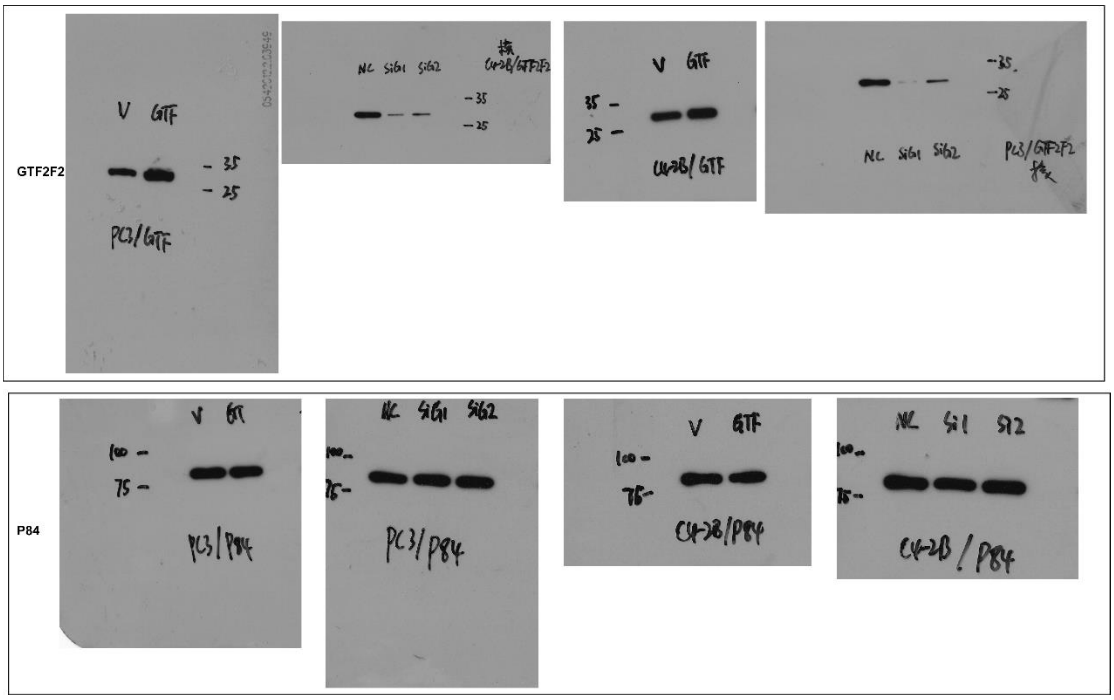

Supplement: Supplementary file 2 — Western blotting datas [file 41420_2023_1585_MOESM2_ESM.pdf]
